# Analysis of 17β-estradiol (E_2_) role in the regulation of corpus luteum function in pregnant rats: Involvement of IGFBP5 in the E_2_-mediated actions

**DOI:** 10.1186/s12958-016-0153-1

**Published:** 2016-04-12

**Authors:** Sudeshna Tripathy, Killivalavan Asaithambi, Jayaram P, Medhamurthy R

**Affiliations:** Department of Molecular Reproduction, Development and Genetics, Indian Institute of Science, Bangalore, 560012 India

**Keywords:** Estradiol, Corpus luteum, IGFBP5, Progesterone, Pregnancy, Rat

## Abstract

**Background:**

In several species, considerably higher levels of estradiol-17 (E_2_) are synthesized in the CL. E_2_ has been suggested to participate in the regulation of luteal steroidogenesis and luteal cell morphology. In pregnant rats, several experiments have been carried out to examine the effects of inhibition of luteal E_2_ synthesis on CL structure and function.

**Methods:**

During days 12–15 of pregnancy in rats, luteal E_2_ was inhibited by way of daily oral administration of anastrozole (AI), a selective non-steroidal aromatase inhibitor, and experiments were also performed with E_2_ replacement i.e. AI+ E_2_ treatments. Luteal tissues from different treatment groups were subjected to microarray analysis and the differentially expressed genes in E_2_ treated group were further examined for expression of specific E_2_ responsive genes. Additional experiments were carried out employing recombinant growth hormone preparation and flutamide, an androgen receptor antagonist, to further address the specificity of E_2_ effects on the luteal tissue.

**Results:**

Microarray analysis of CL collected on day 16 of pregnancy post AI and AI+E_2_ treatments showed significantly lowered *cyp19a1* expression, E_2_ levels and differential expression of a number of genes, and several of them were reversed in E_2_ replacement studies. From the differentially expressed genes, a number of E_2_ responsive genes were identified. In CL of AI pregnant rats, non-significant increase in expression of igf1, significant increase in igbp5, igf1r and decrease in expression of Erα were observed. In liver of AI treated rats, igf1 expression did not increase, but GH treatment significantly increased expression that was further increased with AI treatment. In CL of GH and AI+GH treated rats, expression of igfbp5 was higher. Administration of flutamide during days 12–15 of pregnancy resulted in non-significant increase in igfbp5 expression, however, combination of flutamide+AI treatments caused increased protein expression. Expression of few of the molecules in PI3K/Akt kinase pathway in different treatments was determined.

**Conclusions:**

The results suggest a role for E_2_ in the regulation of luteal steroidogenesis, morphology and proliferation. *igfbp5* was identified as one the E_2_ responsive genes with important role in the mediation of E_2_ actions such as E_2_-induced phosphorylation of PI3K/Akt kinase pathway.

**Electronic supplementary material:**

The online version of this article (doi:10.1186/s12958-016-0153-1) contains supplementary material, which is available to authorized users.

## Background

In several species, the control of corpus luteum (CL) function is broadly accomplished by the dynamic interplay between luteotrophic and luteolytic factors. Of the several luteotrophic factors, three key hormones namely, LH, PRL and E_2_ play critical role and depending on the species, they act to function individually or as a part of the luteotrophic complex to regulate luteal function [20, 28, 36]. Interestingly, all three hormones have been recognised for their trophic actions on structure and function of CL in rats. Studies in rabbit were the first to propose the luteotrophic effects of E_2_ [15]. Expression of *Cyp19a1*gene that encodes the aromatase enzyme responsible for aromatization of androgens into estrogens in the luteal tissue of both pregnant and pseudo pregnant rabbits has been reported [1]. The rat CL is unique in that expression of *Cyp19a1*is highest, and it has been reported that androgens synthesized in placenta are transported to CL for aromatization into E_2_, since placenta lacks *Cyp19a1*expression [19, 40]. Even though the CL of several species is capable of E_2_ biosynthesis and express estrogen receptors ERα and ERβ [3, 33, 34], elucidation of direct effects of E_2_ on CL function has received little attention. Since the rat CL has high capacity for E_2_ biosynthesis, the intraluteal effects of E_2_ will be expected to be pre-eminent. In rats, E_2_ has been reported to have multiple effects on CL function that range from transport of cholesterol for P_4_ biosynthesis, hypertrophy of luteal cells, conversion of small luteal cells into large luteal cells [35, 36]. However, the mechanism/s by which E_2_ mediates these effects in the luteal tissue is poorly understood. In a recent study from our laboratory, differential expression of many E_2_ responsive genes in the luteal tissue was observed during induced luteolysis in two distinct animal models, macaque and the bovine species [32]. In that study, it was observed that following rapid decline in circulating and luteal E_2_ levels, many of the genes belonging to the IGF system were differentially expressed. The components of IGF system which consists of two ligands (IGF1 and IGF2), two receptors and at least six IGF binding proteins (IGFBP1-6) are increasingly being implicated in the control of CL function. In the present study, a number of experiments were carried out to test the hypothesis that E_2_ plays a critical role in the maintenance of luteal function in rats. Inhibition of E_2_ was accomplished by administration of specific aromatase inhibitor (AI)._._In this study microarray analysis was performed to examine differential expression of E_2_ responsive genes with a view to address critical issue of specific effect/action of E_2_ on CL function. The results suggest involvement of IGF system, especially changes in expression of IGF1 and IGFBP5 during E_2_ inhibition and replacement studies. In several species, GH plays a key role in multiple physiological processes largely mediated by increasing IGF1 levels in liver [42]. However, the receptor for GH is expressed in several tissues including the CL [4]. Since IGF1 gene transcription is rapidly and profoundly induced by GH through activation of STAT5b [39], it became to interest to examine the effect of GH on the expression of igf1 in liver and CL during E_2_ inhibition and replacement experiments. Further, we examined whether IGFBP5 participates in mediating E_2_ actions. The results suggest novel roles for IGFBP5 during proliferation and hypertrophy of luteal cells in the control of luteal function.

## Methods

### Reagents

Anastrozole, a selective non-steroidal aromatase inhibitor (AI), 17-β estradiol, testosterone, flutamide and Oil Red O stain were purchased from Sigma-Aldrich Co., Bangalore, India. Recombinant bovine growth hormone (rbGH) was a kind gift from Monsanto Company St. Louis, MO. Oligonucleotide and Oligo dT primers were synthesized by Sigma-Genosys, Bangalore, India. DyNAzyme™ II DNA polymerase was purchased from Finnzymes (Espoo, Finland) and dNTPs were procured from Eppendorf, (Hamburg, Germany). Power SYBR® Green PCR master mix was obtained from Applied Biosystems, Foster City, CA. The details of antibodies employed are provided in Additional file 1: Table S1. The secondary anti-rabbit and the ABC colour development kit was procured from Bangalore Genei, India. Mouse/rat Insulin-like Growth Factor-1 (m/r IGF-1) ELISA kit was procured from BioVendor, Mediagnost, Germany. All other reagents were purchased from Sigma-Aldrich Co., Bangalore, India or sourced from local distributors.

### Experimental protocol, CL and blood collection schedule

*Rattus norvegicus* (Harlan Wistar strain) were housed in a controlled environment and kept under a light: dark cycle of 12 h with ad libitum access to food and water. To obtain pregnant animals, the vaginal smear of the cohabitated females with males was screened daily for presence of sperm and the day of appearance of sperm was designated as day 1 of pregnancy. All procedures in animals were approved by the Institutional Animal Ethics Committee, Indian Institute of Science, Bangalore, India.

#### Experiment 1: *In vitro* aromatisation of testosterone (T) during mid-pregnancy

To determine the activity of aromatase present in the CL tissue and to examine the effectiveness of AI in blocking aromatase activity, *in vitro* studies were performed employing a previously published method [13] with few modifications. CL from day 7, 11, 12 and 16 of pregnant rats were incubated *in vitro* without or with T or AI for examining the aromatization capacity during different days of pregnancy. The individual CL was weighed, sliced into pieces and ~ 10–12 mg pooled tissue/well was used for studies. Tissue samples were placed in wells containing 1 ml M199 containing 10 μl of propylene glycol (VEH) or AI (120 ng/well) without or with T (20 ng/well) and incubated for 4 h at 37 °C with 5 % CO_2_ for determining E_2_ levels in the medium.

#### Experiment 2: Effect of inhibition of luteal E_2_ on structure and function of CL during pregnancy

Experiments were carried out during early (day 7 to 11 of pregnancy) and mid (day 12 to 16 of pregnancy) pregnancies corresponding to low and high E_2_ secreting phases. To determine the suitable dose of AI and duration of treatment required for consistent inhibition of luteal E_2_ synthesis *in vivo*, a pilot study was carried out in which oral administration of various doses of AI (0.1, 0.15, 0.5 and 1 mg/kgBW/day dissolved in a total volume of 0.3 ml of water containing 2 % ethanol) administered on days 7–10 and days 12–15 of pregnancy. The results of pilot study indicated that administration of AI at a dose of 1 mg/kg BW/day on days 12–15 of pregnancy significantly lowered circulating and luteal E_2_ levels on day 16 of pregnancy. Also, the weights of CL were lower and had evidence of loss of implantation.

**Fig. 1:**
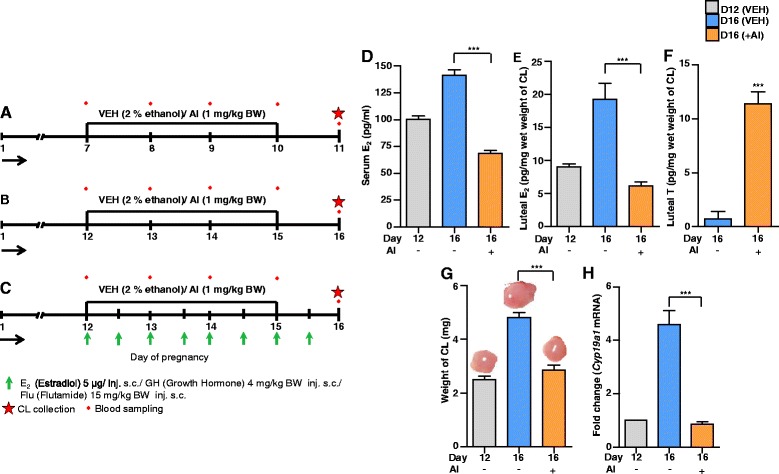
Effects of AI on circulating E_2_, luteal E_2_ and T, weight of CL and *Cyp19a1* mRNA expression during mid pregnancy in rats. Experimental protocols from Experiment 2–5 (A-C) in *Rattus norvegicus*. The CL and blood collection are indicated by a red star and dot, respectively. Pregnant rats received oral administration of VEH (2 % ethanol) or AI (1 mg/kg BW) on day 7 (**a**) or 12 (**b**) for 4 days daily and blood and CL tissue samples were collected on day 11 or 16. **c** Beginning on day 12, pregnant rats received oral administration of AI (1 mg/kg BW) and 5 μg E_2_ or 4 mg/kg BW GH or 15 mg/kg BW Flu/am and pm, s.c. for 4 days daily. CL tissues collected on day 16 of pregnancy. **d** Mean±SEM serum E_2_ concentration during different treatments (*n* = 4 animals/time point, one-way ANOVA, ^***^ *P* <0.001). Two to three corpora lutea were processed for tissue lysate preparation using sterile ice cold 1X PBS for quantitating luteal E_2_ (**e**) and T (**f**) concentrations. Each bar represents mean±SEM, *n* = 4 animals/time point, one-way ANOVA, ^***^ *P* <0.001. **g** Weight of CL during different treatments along with a representative photo for each treatment is shown on each bar (mean±SEM, *n* = 12 CL/time point, one-way ANOVA, ^***^ *P* <0.001). **h** qPCR expression of *Cyp19a1* in CL post different treatments. The results are shown as fold changes in mRNA expression compared with day 12 control. Individual bars represent mean±SEM fold change in mRNA expression value for qPCR analysis during different treatments (*n* = 4 animals/time point, one-way ANOVA, ^***^ *P* <0.001)

To examine the effects of depletion of luteal E_2_ levels on CL structure and function, groups of pregnant rats (*n* = 4 animals/group) were orally administered VEH (2 % ethanol) or AI (1 mg/kg BW) daily on days 7–11 (early pregnancy) and on days 12–15 (mid-pregnancy). The details of experimental protocol, schedule of blood sample collection, treatments and CL collection are represented in Fig. 1a and b. Also, blood samples and CL were collected from a group of untreated rats on day 7 and 12 of pregnancy.

#### Experiment 3: Effect of E_2_ replacement on the function of CL during AI treatment

After confirming of significant inhibition of circulating and luteal E_2_ concentrations and morphological changes in CL post AI treatment (see results), further experiments were carried out to identify the specific effects of E_2_ on CL function and morphology. Groups of pregnant rats (*n* = 4 animals/group) were administered AI+VEH or AI+E_2_ (5 μg) as per the protocol provided in Fig. 1c.

#### Experiment 4: Effect of GH on expression of IGF1 and IGFBP5

Since changes in IGF1 system were observed with AI treatment, studies were conducted to examine GH action on CL function. rbGH was administered at a dose of 4 mg/kg BW s.c. twice daily without (i.e. VEH+GH) or with AI treatment (AI+GH, *n* = 4 rats) during days 12–15 of pregnancy. Blood samples, CL and pieces of liver were collected on day 16 for various analyses as per the protocol provided in Fig. 1c.

#### Experiment 5: Effect of androgen receptor antagonist on the function of CL in AI treated pregnant rats

AI treatment resulted in substantial increase in serum T concentration. To examine whether androgens contributed to changes in CL function, experiments were carried out using androgen receptor antagonist, flutamide (Flu). Flu was administered at a dose of 15 mg/kg BW s.c. twice daily without (i.e. VEH+Flu) or with AI treatment (AI+Flu, *n* = 4 rats) during days 12–15 of pregnancy. Blood samples and CL were collected for various analyses as shown in Fig. 1c.

Corpora lutea from experimental animals were isolated from anesthetized animals for microarray and other analyses. For quantitating tissue E_2_ and T concentration, two or three corpora lutea were processed for tissue lysate preparation using sterile ice cold 1X PBS. For histochemistry, two or three corpora lutea were fixed in NBF solution. The remaining corpora lutea were stored at −20 °C for cryosectioning or flash frozen in liquid nitrogen and stored at −70 °C.

### Hormone assays

Luteal and serum steroids (E_2_ and P_4_) were determined by specific RIAs as reported previously [21, 32]. The sensitivity for E2 and P4 in the assays was 39 pg/ml and 0.1 ng/ml, respectively. The inter- and intra- assay coefficient of variations for both E_2_ and P_4_ hormones were <10 %. The concentration of serum and luteal T was quantitated by a commercially available direct T assay kit (Immunotech, Marseilles, France). The sensitivity of the assay for T was 0.08 ng/ml. The inter- and intra- assay coefficient of variations were 15 and 14.8 %, respectively.

### Plasma IGF1 assay

Plasma IGF1 was quantified using a specific IGF1mouse/rat ELISA kit. The assay utilizes two specific and high affinity antibodies for IGF1. The IGF1in samples bind to the immobilized first antibody on the microtiter plate, the biotinylated and streptavidin-peroxidase conjugated second specific anti-IGF1antibody binds in turn to the immobilized IGF1 To dissociate IGF1 from the IGFBPs, plasma samples were diluted in acidic buffer provided with the kit and the diluted samples were assayed in 50–100 μl as per the manufacturer’s protocol. The sensitivity assay was 0.029 ng/ml. The inter- and intra- assay coefficients of variation were 8.5 and 7.2 %, respectively.

### RNA isolation

Total RNA was extracted from CL and liver tissues from different experiments using TRI Reagent® according to the procedure as reported previously [21].

### cDNA preparation and Semi quantitative RT-PCR analysis

Total RNA was reverse transcribed using the following protocol: 1 μg of total RNA along with 1 μl of Oligo dT was incubated at 65 °C for 10 min and snap chilled on ice for 5 min and 4 μl of 5× RT buffer containing 250 mM Tris HCl (pH 8.3 at 25 °C), 250 mM KCl, 20 mM MgCl_2_ and 10 mM DTT was added followed by 10 mM dNTPs, 20 units of ribonuclease inhibitor, DEPC treated water to make the volume up to 19 μl and to it, 200 units (1 μl) of Revert Aid™ MMuLV Reverse transcriptase was added. The reaction mixture was incubated at 42 °C for 1 h. PCR was carried out using gene specific primers. The efficiency of the RT-PCR was checked using L19 expression, a house keeping gene. For PCR, cDNA equivalent 25 ng of total RNA was used. The PCR mix used in each reaction contained 0.2 mM dNTPs, 2.5 μl of 10× buffer containing 100 mM Tris-HCl pH 8.8 at 25 °C, 15 mM MgCl_2_, 500 mM HCl and 0.1 % Triton X-100; 25 μM each of forward and reverse primers and 1 unit of DyNAzyme™ II DNA polymerase. For selecting the annealing temperature, the temperature gradient semi quantitative RT-PCR was performed as reported previously [21].

### qPCR analysis

The analysis was carried out essentially as described previously [29, 32]. The diluted cDNA samples equivalent to 10 ng of total RNA were subjected to validation analysis on Applied Biosystems 7500 Fast Real Time PCR system with SDS v 1.4 program employing Power SYBR green 2× PCR master mix. The 10 μl qPCR mixture contained cDNA equivalent to 10 ng of total RNA, 5 μl of PCR master mix and 5 μM each of forward and reverse gene specific primers. PCRs were carried out in duplicates in 96 well plates. The initial enzyme activation was carried out at 95 °C for 10 min, denaturation was carried out at 95 °C for 30 s, the annealing was carried out at specific annealing temperature for 30 s and extension was at 72 °C for 30 s with a final extension of 5 min at 72 °C. Analysis of expression of each gene included a no template control (NTC) and generation of a dissociation curve. Expression levels of the genes validated were normalized by using L19 expression levels as calibrator or internal control for each cDNA sample. Primers were designed using rat (*Rattus norvegicus*) sequences submitted at NCBI and ENSEMBL using Primer Express™ version 2.0 (Applied Biosystems, Foster City, CA, USA). The primers were designed to cover the exon-exon junctions. The details of primers employed along with the amplicon size and annealing temperature are provided in Additional file 2: Table S2. Real time PCR efficiencies were acquired by amplification of a standard dilution series (with 10 fold differences) in the Applied Biosystems 7500 Fast Real time PCR system with SDS v 1.4 program employing Power SYBR Green 2X PCR mix. The corresponding efficiencies (E) for different gene primers were calculated according to the equation: E = 10^[−1/slope]^ −1 [25] and an efficiency of >90 % was obtained for all. Analysis of expression of each gene included a no template control (NTC) and generation of a dissociation curve. Expression levels of the genes validated were normalized by using L19 expression levels as calibrator (internal control) for each cDNA sample. The relative expression and fold change in gene expression was determined using DC_t_ and DDC_t_ method, respectively. Relative expression = 2^-DCt^ and fold change = 2^-DDCt^, where C_t_ = Threshold cycle i.e. the cycle number at which the relative fluorescence of test samples increases above the background fluorescence, DC_t_ = [C_t_ gene of interest (unknown sample) - C_t_ of L19 (unknown sample)] and DDC_t_ = [C_t_ gene of interest (unknown sample) - C_t_ of L19 (unknown sample)] - [C_t_ gene of interest (calibrator sample) - C_t_ of L19 (calibrator sample)]. PCR for each sample was set up in triplicates and the average C_t_ value was used in the DDC_t_ equation.

### Microarray target preparation, hybridization and analysis

RNA samples from CL of animals of day 12 and day 16 (VEH, AI and AI+E_2_ treatment groups) of pregnancy were subjected to microarray analysis. Three Affymetrix Rat Gene 1.0 ST Arrays [transcript (gene) version] i.e. three RNA samples from individual animals/group were used.

The detailed description of procedures and subsequent generation of processed image files of microarray analysis reported previously for other species [29, 32] were followed for this study. The microarray procedure and data analysis were performed as per Minimum Information About Microarray Experiments (MIAME) compliance. The raw data and the completed analysis of microarray data files have been deposited at NCBI’s Gene Expression Omnibus (GSE41735). ‘R’ software version 2.12.2/Bioconductor (FHCRC labs, Seattle, WA) was used for RMA normalization and for identification of differentially expressed transcripts. The statistical analysis employed for analysing the differentially expressed genes was essentially similar to the recently published work from the laboratory [32]. The data analyzed by Bioconductor analysis tool employing ≥2.0 fold change (except for identification of E_2_ target genes, in which changes >1.5 fold was considered for analysis) cut off and statistical filters provided a number of differentially expressed genes and those found common between different treatments (VEH, AI and AI+E_2_ treatments). For validation of microarray analysis, eight genes were selected for qPCR analysis. The statistically significant (*P* <0.05) correlation between the two analyses was determined as reported previously [32]. The differentially expressed genes were clustered by hierarchy analysis by GeneSpring analysis for all the probe sets of each treatment group is represented as dendrograms (data not shown). To examine molecular function and genetic networks, microarray data was analyzed using Ingenuity Pathway Analysis (IPA version 8.7, Ingenuity® Systems Inc., Redwood City, CA; http://www.ingenuity.com).

### Analysis of expression of E_2_ responsive genes

To examine the role of E_2_ in the regulation of rat CL structure and function, the differentially expressed genes identified in CL of E_2_ inhibition (AI) and replacement (AI+E_2_) groups were mined for E_2_ responsive genes. For this purpose, 89 genes were chosen as E_2_ responsive genes and the list of genes was same as previously reported for another study relating to macaque and bovine CL [32].

### Immunoblot analysis

CL tissue lysate preparation and immunoblot analysis were carried out as per the previously published procedures [24].

### Histology

Immunohistochemistry for ki67 was carried out on a piece of ovary containing 2–3 CL as described previously [43].

### Oil Red O staining

Pieces of ovaries containing at least 2–3 CL stored at −20 °C were subjected to cryotome sectioning (~8 μm thickness) and fixed in 10 % formalin for 5–10 min. The sections were immersed in absolute propylene glycol for 2–5 min followed by staining in 60 °C pre heated Oil Red O stain for 8–10 min. The slides were differentiated in 85 % propylene glycol for 2–5 min, washed in distilled water and stained with hematoxylin before mounting with glycerine jelly for observation under inverted microscope.

### Statistical analyses

The hormone data, qPCR fold change expression data among different experimental groups and densitometric data are expressed as mean±SEM. For multiple comparisons, the data were analyzed by one-way ANOVA, followed by Newman-Keuls multiple comparison test (PRISM Graph Pad, version 5; Graph Pad Software, Inc., San Diego, CA). Comparisons between mean of two groups were carried out using Student t-tests. A *P* value of <0.05 was considered to be statistically significant.

## Results

### Analysis of *in vitro* aromatization in CL tissue

The results indicated that although basal E_2_ secretion from CL tissue slices on different days did not change, but addition of T resulted in significant increase in E_2_ secretion. Furthermore, addition of AI had no effect on basal E_2_ secretion, but inhibited T conversion to E_2_ from tissue slices of day 16 pregnancy (Additional file 3: Figure S1A).

### AI treatment on CL function during early pregnancy

Circulating E_2_ levels on day 11 following AI treatment on days 7–10 of pregnancy was 68.2 ± 3.89 pg/ml which was significantly lower (*P* <0.05) compared to VEH treated animals (82.8 ± 3.98 pg/ml, Additional file 3: Figure S1B). Circulating P_4_ levels (64.5 ± 2.72 vs. 70.0 ± 1.78 ng/ml), CL weight (2.19 ± 0.12 vs. 2.16 ± 0.09 mg/CL) and *Cyp19a1* mRNA expression (Additional file 3: Figure S1C-E) were not significantly different between AI and VEH treated animals. Based on these findings, further studies employing AI treatment were not carried out during early pregnancy.

### AI treatment on CL function during mid pregnancy

**Fig. 2:**
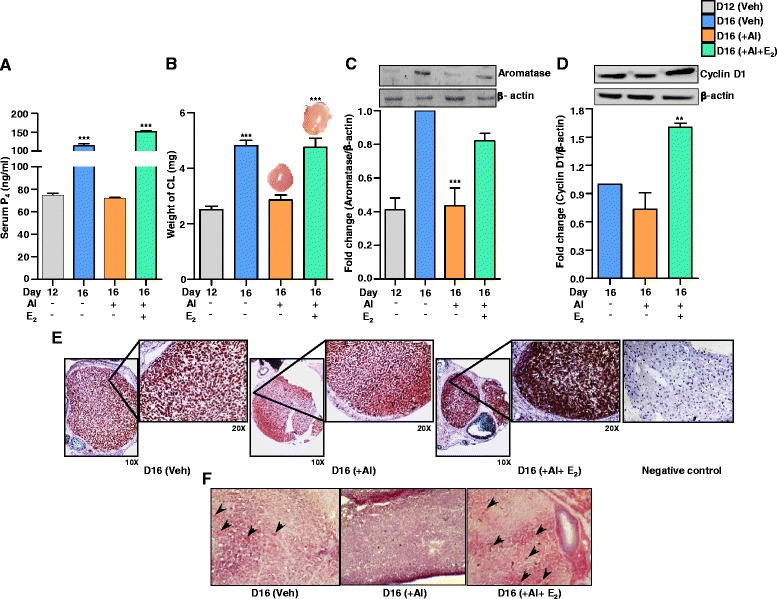
Effects of luteal E_2_ inhibition and replacement of E_2_ on serum P_4_, CL weight, aromatase expression, cyclin D1, ki67 and luteal lipid droplets content. Beginning on day 12, pregnant rats received daily oral administration of AI (1 mg/kg BW) and 5 μg E_2_ (am and pm), s.c. for four days. CL tissues collected on day 16 of pregnancy were subjected to microtome and cryosectioning. **a** Circulating mean serum P_4_ concentrations during different treatments (*n* = 4 animals/time point, one-way ANOVA, ***P <0.001). **b** Weight of CL during different treatments with representative photo is shown for AI and AI+E_2_ treatments. Each bar represents mean±SEM, *n* = 12 CL/time point, one-way ANOVA, ^***^ *P* <0.001. Protein levels of aromatase (**c**) and cyclin D1 (**d**). Protein lysate (50 μg) prepared from CL tissue from different treatments were resolved on 10–12 % SDS PAGE, transferred onto PVDF membrane and immunoblot analysis was performed using antibodies against aromatase, cyclin D1 and β-actin. A representative immunoblot for each antibody probed is shown. The immunoblot probed with β-actin antibody was used as loading control for each lane. Each bar represents the fold change (mean±SEM; *n* = 4 animals/time point) with respect to day 16 VEH treated CL, relative to intensity of β-actin for each treatment (one-way ANOVA, ^***^ *P* <0.001, ^**^ *P* <0.01). **e** Photomicrographs represent ki67 immunostaining of CL tissue sections post different treatments. Samples were counter stained with haematoxylin, and images taken at a magnification of 20×. For reference, an image for the negative control is shown. **f** Representative image of cryosectioned CL tissue sections for oil red O staining across different treatments. *Arrow heads* indicate cells stained for lipid content

Serum P_4_ concentration was significantly higher on day 16 VEH treated group (113.75 ± 5.54) compared to day 12 (74.5 ± 2.22) pregnant rats (Fig. 2a). The P4 concentration did not increase in AI treated group (Fig. 2a). Serum E_2_ concentration decreased significantly after AI treatment [141.25 ± 5.15 (VEH) vs. 68.5 ± 2.53 (AI) pg/ml, Fig. 1d]. Further, luteal E_2_ levels were significantly lower in AI treated animals [6.15 ± 0.58 pg/mg CL vs. 19.27 ± 2.43] compared to VEH treated group, Fig. 1e, while luteal T levels were significantly higher (11.37 ± 1.14 pg/mg CL) post AI treatment (Fig. 1f). AI treatment caused a significant decrease in CL weight (2.86 ± 0.17 vs. 4.82 ± 0.18 mg/CL for AI and VEH treated groups, respectively, Fig. 1g) and decrease in *Cyp19a1* mRNA expression (Fig. 1h).

### Replacement of E_2_ during AI treatment

Co-administration of E_2_ during AI treatment on day 12–15 of pregnancy significantly increased P_4_ concentration (Fig. 2a). The weight of CL in AI+E_2_ treated animals (4.75 ± 0.32 mg/CL) was not significantly different from the VEH treated group (4.82 ± 0.18 mg/CL), but higher than AI+VEH treated group (Fig. 2b). The aromatase protein level was low in CL of day 12 pregnancy, but was higher in CL of day 16 VEH treated group (Fig. 2c). AI treatment significantly decreased the aromatase level compared to day 16 VEH treated group (Fig. 2c). Co-administration of E_2_ during AI treatment prevented the inhibition of aromatase level (Fig.2c; luteal E_2_ levels 21.5 ± 1.56 pg/mg CL). Cyclin D1 levels were low on day 16 pregnancy and day 16 AI treated group, but was higher in AI+E_2_ treated group (Fig. 2d). To further determine E_2_ effect on cell proliferation, the cell proliferation marker, ki67 expression was examined in the CL. The intensity of expression was higher in CL of AI+E_2_ treated animals (Fig. 2e). The lipid content of luteal cells was examined by oil red O staining and more lipid droplets could be visualized in AI+E_2_ treated group (Fig. 2f).

### Microarray analysis of CL

**Fig. 3:**
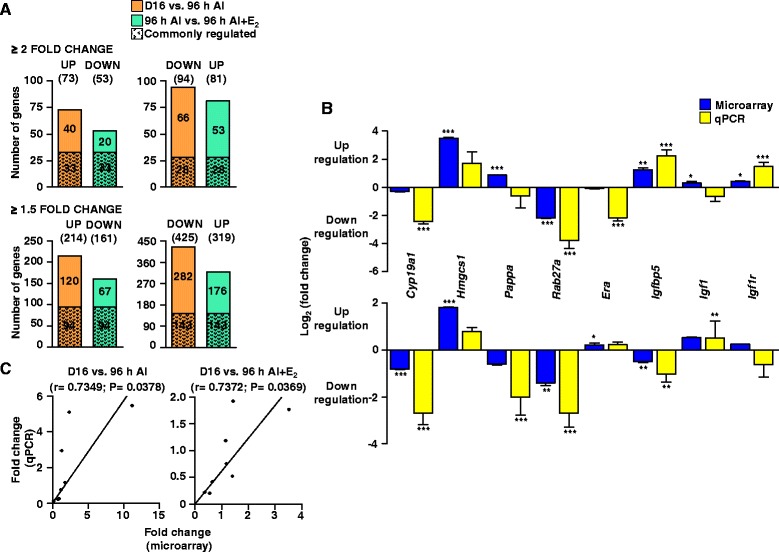
Schematic representation of temporal changes in differentially expressed genes and validation of microarray data of select genes by qPCR analysis. **a** Bar graphs representing the number of differentially expressed genes identified after microarray analysis of CL tissues collected from rats treated with VEH, AI & AI+E_2_ treatments. Data analyzed by Bioconductor analysis tool employing ≥2 and ≥1.5 fold change cut-offs. A comparison of total number of up regulated and down regulated genes between VEH vs. AI (*orange bars*) and vs. AI+E_2_ (*green bars*). The commonly regulated genes post E_2_ deprivation whose expression reversed with E_2_ replacement is represented by filled bars. **b** The fold change expression (microarray data) of selected up and down regulated genes associated with specific biological process at AI and AI+E_2_. These genes were also subjected to qPCR analysis and log_2_ (fold change) expression. Individual bars for each gene represents mean±SEM values in mRNA expression for microarray analysis (*blue bars*) and qPCR analysis (*yellow bars*) during different treatments (*n* = 3 for microarray analysis and *n* = 4 for qPCR analysis. *Upper panel*, VEH vs. AI and *lower panel*, VEH vs. AI+E_2_. ^***^ *P* <0.001, ^**^ *P* <0.01, ^*^ *P* <0.05). Individual bars for each gene represents mean±SEM values in mRNA expression for microarray analysis (*blue bars*) and qPCR analysis (*yellow bars*) during different treatments (*n* = 3 for microarray analysis and *n* = 4 for qPCR analysis. ^***^ *P* <0.001, ^**^ *P* <0.01, ^*^ *P* <0.05). **c** Correlation analysis between expression ratios obtained from microarray and qPCR analyses of few genes post different treatments (*n* = 8 genes/treatment). Linear regression analysis was performed for selected differentially expressed genes using qPCR fold change in expression values (2^-ΔΔCT^; Y-axis) with fold change expression values obtained by microarray analysis (X-axis). The r value, correlation coefficient, generated for the theoretical line of best fit (represented as *solid line* in each panel) and the *P* value indicates significance of the correlation as determined by ‘F’ test

The analysis at two fold cut off identified differential expression of 167 genes (73 and 94 genes, up and down regulated genes) in CL of AI treated rats. Following AI+E_2_ treatment, differential expression of 134 genes (81 and 53 up and down regulated genes) were identified (Fig. 3a). The differential expression of 61 genes identified in AI treated group was reversed in AI+E_2_ treated group. The list of differentially expressed genes in the microarray data with >1 fold cut off was used with a view to select maximum number of differentially expressed E_2_ responsive genes for purpose of carrying out pathway analysis. The number of up and down regulated genes and the number of commonly regulated genes between AI and AI+E_2_ treated groups are presented in Fig. 3a. From the differentially expressed genes, eight genes were subjected to qPCR analysis to validate the microarray data and the results are presented in Fig. 3B. The goodness of fit analysis of the data yielded a good correlation coefficient ‘r’ values for AI [0.7349 (*P* = 0.0378)] and AI+E_2_ [0.7372 (*P* = 0.0369)] treatments which suggested that qPCR data corroborated well with the microarray data (Fig. 3c).

### Pathway analysis of microarray data

**Fig. 4:**
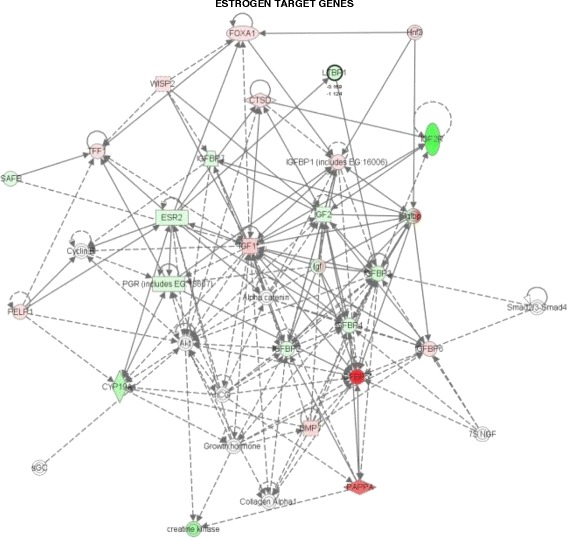
Classification of differentially expressed genes into E_2_ target genes by Ingenuity Pathway Analysis (IPA). IPA of the differentially regulated genes post AI treatment shows a network of 26 focus molecules. The network is displayed graphically as nodes (gene/gene products) and edges (biological relationship between nodes). They are represented by different shapes with their expected regulation of a particular cellular or biological function. The red and green nodes represent up and down regulated genes or their products. The intensity of node colour indicates the abundance and various shapes of the nodes represent molecular class of the gene product. The solid lines indicate interaction of molecules being discussed that have been validated through various experiments and reported in literature. On the other hand, the dashed lines are tentative interaction which are not yet completely validated and are based on computationally designed programs

The ingenuity pathway analysis (IPA) software was used to classify the differentially expressed genes (1.5 fold cut off) into different function and disease categories. The IPA analyses revealed that 85–90 % of the differentially expressed genes were function or pathway eligible. Nine networks were identified with score and number of focus molecules ranging from as low as one to as high as 22. The details of different networks for AI and AI+E_2_ treatment groups are highlighted in Additional file 4: Table S3 and Additional file 5: Table S4. The IPA software analysis indicated that six networks (network #1, 2, 3, 5, 6 and 8) for VEH vs. AI treatment and four networks (network #2, 3, 7 and 8) for AI vs. AI+E_2_ treatment were found to overlap with each other directly or indirectly. The network selection was further categorized into differentially regulated genes associated with E_2_ target (Fig. 4), steroidogenesis (Additional file 6: Figure S2A) and growth factors (Additional file 6: Figure S2B) with 26, 26 and 16 focus molecules, respectively. The findings emphasize the role for E_2_ in the regulation of CL function.

### Expression of E_2_ responsive genes in the pregnant CL

**Fig. 5:**
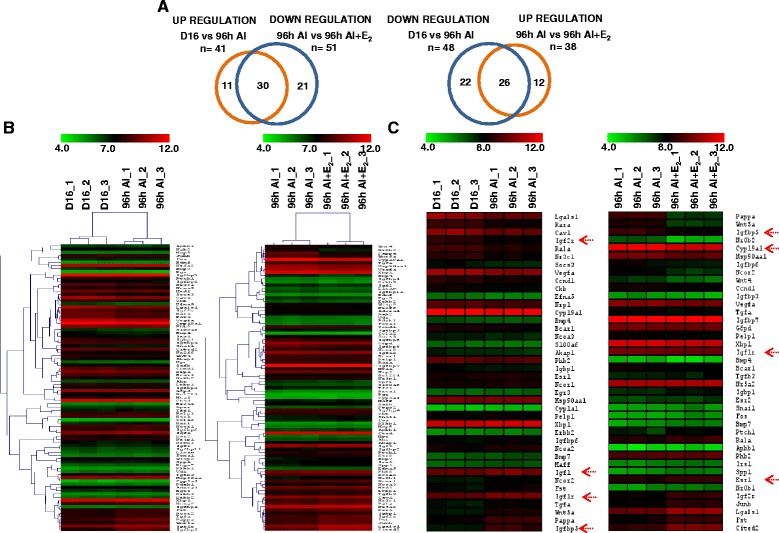
Schematic representation and dendrogram generated after hierarchical clustering of differentially expressed E_2_ responsive genes during different treatments. **a** Venn diagrams representing the number of differentially expressed genes post different treatments. Data was analyzed by Bioconductor analysis tool. Thirty and 26 genes that were identified as up and down regulated after E_2_ inhibition that were identified as down and up regulated with E_2_ replacement treatment. A comparison of total number of up and down regulated genes was done across AI (*orange*) and AI+E_2_ (*blue*). **b** Heat map or combined tree of the hierarchy analysis (by clustering gene identity and conditions) for probe sets at each time point. i.e. VEH vs. AI and AI vs. AI+E_2_. The plot includes two dendrograms- vertical dendrogram for gene identity, and horizontal for different treatments. A visual dual colour code, red and green, is utilized to indicate relatively up or down regulation, respectively. Top of each dendrogram shows the condition colour bar with the parameters in each interpretation. **c** Top 40, up and down regulated genes from the analysis were utilized to construct heat map to get a view of the differentially regulated E_2_ regulated genes post different treatments. The top of each dendrogram shows the condition colour bar. Hovering over the cells of the heat map, the normalized intensity values are represented in various shades of red and green indicating relatively either up or down regulation, respectively. The row header shown on the right side represents the complete entity name or gene symbol. The genes *Cyp19a1, Igfbp5, Igf1, Igf1r,* and Esr1 are further highlighted with *red arrows*

All 89 genes that were regarded as E_2_ responsive genes were identified to be differentially expressed post AI and AI+E_2_ treatments. There were 41 (up) and 48 (down) genes differentially expressed post AI treatment. Following E_2_ replacement, 38 (up) and 51 (down) E_2_ responsive genes were differentially expressed compared to AI treated group. Venn diagrams were constructed to highlight those genes whose expression pattern was reversed in CL of animals receiving AI+ E_2_ treatment (Fig. 5a). As many as 56 genes [30 (up) and 26 (down)] were found to have their expression pattern reversed in AI+E_2_ treated rats (Fig. 5a). The initial analysis highlighted a set of authentic E_2_ responsive genes and a few of these genes were selected for further analysis. The list of top 15 up and down differentially expressed genes regarded as E_2_ responsive genes both in AI and AI+E_2_ treatment groups are provided in Additional file 7: Table S5 and Additional file 8: Table S6.

### Hierarchical clustering of E_2_ regulated genes

A dendrogram (heat map) view produced by hierarchical clustering of E_2_ regulated genes from AI and AI+E_2_ treatments is represented in Fig. 5b. Further, top 40 differentially expressed E_2_ responsive genes were used to construct heat map as in Fig. 5c. The differentially expressed genes belonging to IGF system were selected for further studies.

### Effects of AI and AI+E_2_ treatment on the IGF system in the CL

**Fig. 6:**
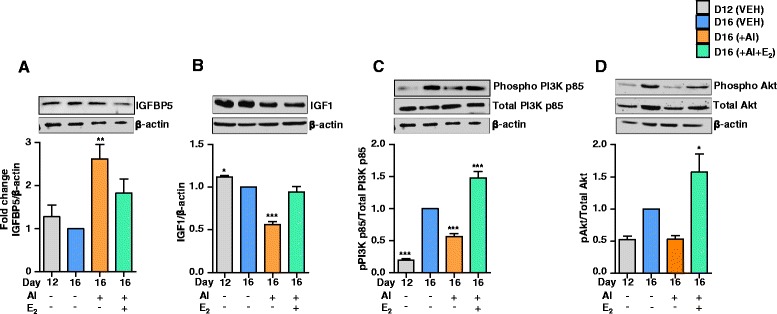
Effects of E_2_ inhibition and replacement on protein levels of IGFBP5, IGF1, pPI3K p85, Total PI3K p85, pAkt and Total Akt. Day12 pregnant rats received oral administration of AI (1 mg/kg BW) and 5 μg E_2_ (am and pm) s.c. twice daily. Protein levels of IGFBP5 (**a**), IGF1 (**b**), pPI3K p85 and Total PI3K p85 (**c**), and pAkt and total Akt (**d**). Protein lysates (50 μg) prepared from CL tissue from different treatments were resolved on 10/12 % SDS PAGE, transferred onto PVDF membrane and immunoblot analysis was performed using antibodies specific to each protein. A representative immunoblot for each of the antibody probed is shown. The immunoblot probed with β-actin antibody was used as loading control for each lane (same immunoblot was shown for panel A&C, since IGFBP5 and pPI3K p85 and total PI3K p85 were probed on the same blot). Each bar represents the fold change with respect to day 16 VEH and indicated as mean±SEM (*n* = 4 animals/time point), relative to intensity of β-actin for each treatment (one-way ANOVA, ^***^ *P* <0.001, ^**^ *P* <0.01, ^*^ *P* <0.05). For pPI3K p85 and pAkt, densitometric values were determined relative to intensity of Total PI3k p85 and total Akt, respectively

Figure 6 shows protein levels for IGFBP5, IGF1, pPI3K p85 and pAKT in the luteal tissue of AI and AI+E_2_ treated rats. IGFBP5 levels increased (Fig. 6a) and IGF1 levels decreased (Fig. 6b) in AI treated rats, but these changes were reversed following co-administration of E_2_ with AI (Fig. 6a and b). Analysis of signalling molecules downstream of both E_2_ and IGF signalling revealed that AI treatment significantly lowered IGF1 (Fig. 6b), pPI3K p85 (Fig. 6c) and non-significantly decreased pAKT levels (Fig. 6d) compared to VEH treatment, but these effects were reversed in AI+E_2_ treated rats (Fig. 6b-d). The distinct changes that follow post E_2_ inhibition in the CL tissue may be triggered by up regulation of IGFBP5 levels and accompanying decrease in the IGF and ER signalling (E_2_-ER/PI3K-Akt cascade). The results suggest a key role for E_2_ and its regulation of IGF signalling in the luteal tissue.

### Plasma IGF1 levels and IGF1 expression in liver and CL tissues

**Fig. 7:**
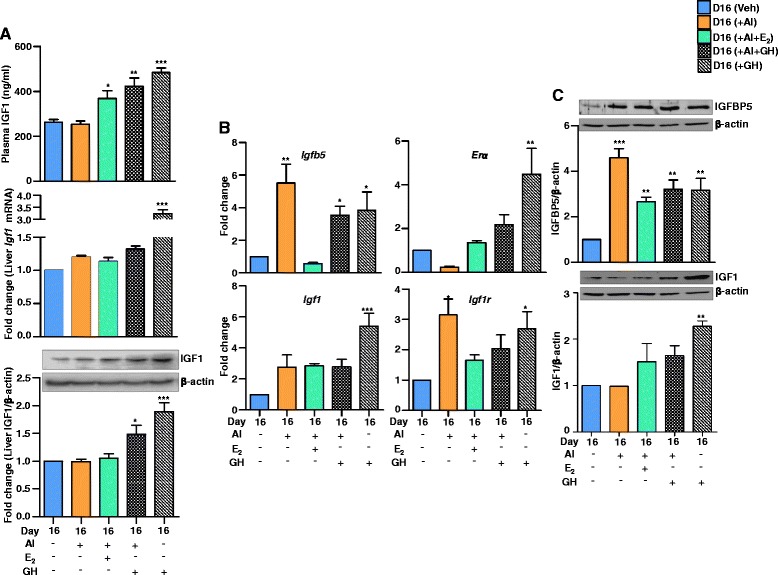
Circulating IGF1 levels and expression of IGF signalling molecules in liver and CL during different treatments. Day12 pregnant rats received oral administration of VEH (2 % ethanol)/AI (1 mg/kg BW), 5 μg (am and pm) E_2_ s.c. and 4 mg GH/kg BW (am and pm), s.c. daily for four days. **a** Upper panel, circulating plasma IGF1 concentrations during different treatments (*n* = 4 animals/time point). Individual bar represents mean±SEM plasma IGF1 concentrations during different treatments, one-way ANOVA, ^***^ *P* <0.001, ^**^ *P* <0.01, ^*^ *P* <0.05. **a** Middle panel, qPCR analysis of Igf1 mRNA. The results are shown as fold changes of mRNA expression during treatments compared to day 16 VEH. Each bar represents mean±SEM, one-way ANOVA, ^***^ *P* <0.001. **a** Lower panel, a representative immunoblot analysis of IGF1. β-actin was used as loading control for each lane. Each bar represents the fold change with respect to VEH treated liver and indicated as mean±SEM (one-way ANOVA, ^***^ *P* <0.001, ^*^ *P* <0.05 *n* = 4 animals/time point) relative to intensity of β-actin for each treatment. **b** qPCR fold change expression of *Igfbp5*, *Erα*, *Igf1* and *Igf1r* mRNA from different treatment groups. The fold expression change for VEH was set as 1 fold change. Individual bars for each gene represents mean±SEM fold change in mRNA expression for qPCR analysis during different treatments (*n* = 4, one-way ANOVA, ^***^ *P* <0.001, ^**^ *P* <0.01, ^*^ *P* <0.05). **c** A representative immunoblot analysis for IGFBP5 and IGF1 in CL during different treatments. β-actin was used as loading control for each lane. Each bar represents the fold change with respect to VEH treatment and indicated as mean±SEM (*n* = 4 animals/time point), relative to intensity of β-actin for each treatment (one-way ANOVA, ^***^ *P* <0.001, ^**^ *P* <0.01)

Treatment with AI had no effect on plasma IGF1 levels, whereas GH treatment significantly increased IGF1 levels and also in rats receiving AI+E_2_ treatment (Fig. 7a). A combination of AI+E_2_+GH increased IGF1 levels but not different from rats receiving GH alone (Fig. 7a). In the liver, IGF1 mRNA expression was higher in GH treated rats and the protein levels were higher in both GH and AI+GH treated rats (Fig. 7a), but no change in IGF1 mRNA expression and protein levels were observed in AI and AI+E_2_ treated rats (Fig. 7a).

### Analysis of expression of genes associated with IGF signalling during different treatments

Since changes in expression of *Igfbp5*, *Erα* and *Igf1r* in CL of AI and AI+E_2_ treated rats were observed, effects of GH on expression of these genes were examined. GH treatment did not lead to significant increase in P_4_ levels (110.8 ± 10.4 ng/ml) on day 16 of pregnancy compared to VEH treated rats (105.2 ± 8.5 ng/ml). Administration of combination of AI+GH treatments resulted in decreased P_4_ concentrations (68.4 ± 6.2 ng/ml). The luteal weights were 3.9 ± 0.22 mg/CL and 3.4 ± 0.2 mg/CL following GH and AI+GH treatments compared to weight of 4.64 ± 0.12 mg/CL from VEH treated rats. GH treatment increased *Igfbp5* expression in CL (Fig. 7b), but combination of AI and GH did not further increase the *Igfbp5* expression (Fig. 7b). The *Erα* expression was high in CL of AI+GH treated rats, but was significantly higher in rats receiving GH treatment. Figure 7b shows expression of *Igf1* and *Igf1r* mRNA in CL of rats receiving VEH, AI, AI+E_2_, AI+GH and GH alone treatments during days 12–15 of pregnancy. The *Igf1* mRNA expression was significantly higher only in GH treated rats compared to all other treatments (Fig. 7b). Although *Igf1r* expression in CL was lower in AI+E_2_ treated rats, but the expression remained high in AI+GH and GH alone treated rats (Fig. 7b).

The protein levels of IGFBP5 and IGF1 in VEH treated rats were set as 1 fold and levels in other treatment groups were expressed in relation to the VEH treatment. The IGFBP5 levels increased significantly in all treatment groups (Fig. 7c). In the CL, IGF1 protein level did not increase with AI treatment, but significantly increased with GH treatment and was non-significantly higher in AI+E2 and AI+GH treated rats (Fig. 7c). However, in the liver tissue IGF1 levels were highest in GH treated rats, but was also higher in AI+E_2_ treated rats (Fig. 7a).

### Effect of flutamide (Flu) on CL function

**Fig. 8:**
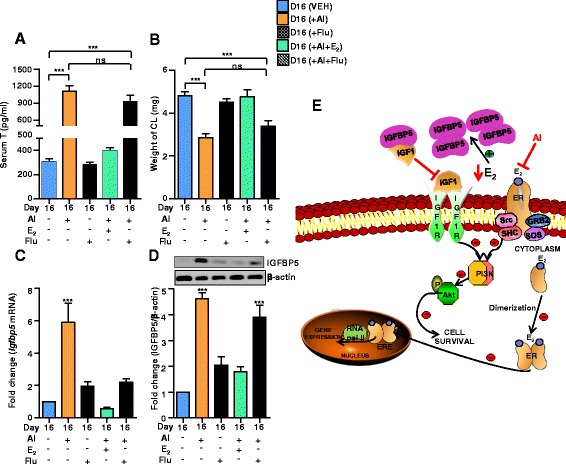
Effects of luteal E_2_ inhibition, replacement and androgen receptor antagonist on serum T, CL weight, IGFBP5 mRNA and protein levels. **a** and **b** circulating mean±SEM serum T concentrations and weight of CL during different treatments (*n* = 4 animals/time point and *n* = 12 CL/time point for weight calculation one-way ANOVA, ^***^ *P* <0.001). **c** qPCR expression analysis of *Igfbp5* in CL post different treatments. The results are shown as fold changes of mRNA expression compared with day 16 VEH group. Individual bars represents mean±SEM fold change during different treatments (*n* = 4 animals/time point, one-way ANOVA, ^***^ *P* <0.001). **d** Immunoblot analysis of IGFBP5. A representative immunoblot is shown. β-actin was used as loading control for each lane. Each bar represents the fold change with respect to day 16 VEH and indicated as mean±SEM (*n* = 4 animals/time point), relative to intensity of β-actin for each treatment (one-way ANOVA, ^***^ *P* <0.001). **e** Schematic representation of IGFBP5 modulation of classical IGF and E_2_ activated signalling pathways in the rat luteal cell. Increased IGFBP5 levels as a result of E_2_ inhibition by AI causes decrease in circulating IGF1 levels leading to decreased activation of IGF1R (a tyrosine kinase) resulting in decreased activation of PI3K and pAkt/PKB levels. Simultaneously, due to unavailability of E_2_ leads to inhibition of PI3K/Akt signalling pathway and thus inhibiting the survival pathway. *E*_*2*_ 17β-estradiol, *ER* estrogen receptor, *IGFBP5* insulin- like growth factor binding protein 5, *IGF1* insulin like growth factor 1, *IGF1R* IGF1 receptor, *Src* non receptor protein tyrosine kinase, *SHC* Src homology 2 domain containing transforming protein, *GRB2* growth factor receptor bound protein 2, *SOS* Son of sevenless, PI3K, phosphatidylinositol 3-kinase, *Akt* protein kinase B, *p* phosphate group, *ERE* estrogen response element, *AI* aromatase inhibitor, *ns* non-significant

AI treatment caused increased circulating T concentration. Employing androgen receptor antagonist, Flu, experiments were carried out to examine whether increased androgen concentration contributed to changes in CL weight and function. Serum T concentration for various treatments are presented in Fig. 8a. Treatment with AI resulted in significantly higher concentration of T compared to VEH treatment (Fig. 8a). Co-administration of Flu with AI treatment did not significantly alter T concentration compared to AI treated rats. The weight of CL in Flu treated rats was not significantly different from the VEH treated rats [4.82 ± 0.18 (VEH) vs. 4.51 ± 0.16 mg/CL (Flu)]. The mRNA expression of IGFBP5 was not significantly higher compared to its expression in CL of VEH treated rats (Fig. 8c). Surprisingly, increased expression of IGFBP5 seen in AI treated rats was not observed in AI+Flu treated rats (Fig. 8c). The protein levels of IGFBP5 in CL of Flu treated rats were high in AI+Flu treated rats, but was not statistically significant from the AI treated group (Fig. 8d).

## Discussion

In rats, the luteal maintenance of P_4_ synthesis during the second half of pregnancy is a complex one involving several players, of which E_2_ is regarded as an important component. In the present study, the findings of P_4_ secretion pattern in AI and AI+E_2_ treated rats were largely in accordance with observations reported previously by others employing hypophysectomized and hysterectomized rat model system [11]. It should be pointed out that [11] employed a very high dose of E_2_ in contrast to the very low dose of E_2_ used in the present study that was devoid of detrimental effects on the implanted embryos. Nonetheless, the observation that E_2_ supplementation stimulated P_4_ secretion in the present study confirms the findings of others [10, 16]. The reported decreased luteal weight observed in hypophysectomized and hysterectomized pregnant rats [9] was also observed in AI treated rats in the present study, however co-administration of E_2_ with AI treatment restored the luteal weight to VEH treated rats. Gibori & Sridaran [9] suggested that the decreased luteal weight in the hypophysectomized and hysterectomized pregnant rats was due to atrophy and that E_2_ administration restored luteal weight without causing changes in cell number. However, based on the results of the present study, we suggest that in addition to atrophy, the cell numbers would also be affected due to E inhibition, since markers of cell cycle and cell proliferation (Cyclin D1 and ki67) were observed to be lower in CL of AI treated rats, but were higher in AI+E_2_ treated rats. However, it remains to be determined whether E_2_ inhibition following AI treatment resulted in increased incidence of apoptosis. Moreover, the weight of CL increases remarkably in size throughout gestation in rats [14], which further suggests that growth of the luteal tissue involves increase in size as well as number of luteal cells. This conclusion is further supported by observations that E_2_ besides being mitogen, also functions as survival factor by way of activation of PI3K-Akt kinase pathway, and E_2_ lack has been shown to cause apoptosis [18, 30]. The results of E_2_ inhibition and E_2_ replacement experiments suggest that E_2_ is critical to CL function in pregnant rats.

To date, several expression profiling studies carried out employing different aromatase inhibitors have been reported for breast cancer lines and ovarian tissues [8]. However, this is the first study detailing global transcriptome changes observed in CL following inhibition of aromatase expression by systemic administration of aromatase inhibitor. In the present study, the microarray analysis data revealed several differentially regulated genes associated with pathways related to steroidogenesis, adipogenesis, cell growth, differentiation and apoptosis. Further, the microarray data of CL from E_2_ inhibited and E_2_ replaced animal models were utilized for identifying differentially expressed E_2_ responsive genes. Surprisingly, expression of number of E_2_ responsive genes was found to be affected by inhibition of luteal E_2_ and the expression of many of these genes was reversed in the E_2_ replacement study. Recently, we reported a list of differentially expressed E_2_ responsive genes during the early time points of luteolysis in two distinct species whose CL are considered to synthesize E_2_ either sparsely (cows) or more abundantly (macaques) [32]. In that study, although the luteolysis was induced by different methods in both the species, surprisingly a rapid decline in luteal E_2_ was observed and that was accompanied by differential expression of a large number of E_2_ responsive genes and several of those genes were also differentially expressed in the CL of pregnant rat. Based on these observations it can be concluded that E_2_ plays an important role in the regulation of luteal function, however, it should be pointed out that participation of luteotrophic and luteolysis factors in each species is distinct from the other and those factors largely determine the effect of E_2_ on the luteal function. The studies on E_2_ inhibition and replacement experiments suggest differential expression of several E_2_ responsive genes and many of them were identified as members of IGF system. The differential expression of IGFBP5 was further examined with different treatments. Surprisingly, GH also caused increased expression of IGFBP5 in the CL tissue.

In the present study, few of the molecules associated with the PI3K signalling pathway were examined. Although the downstream targets of PI3K are many, but Akt is regarded as the primary mediator of PI3K regulating cellular component that affect cell survival [31]. Our findings that mechanisms associated with cell survival, progression, etc., were affected due to E_2_ inhibition further strengthens the hypothesis that E_2_ plays an important role in the regulation of CL function. The presence of androgen receptors in the rat CL and their activation has been reported to be associated with inhibition of apoptosis [16]. The incidence of apoptosis was not examined in the present study; instead luteal weight, steroid hormone levels as well as expression of IGFBP5 were determined. In so far as the protein level of IGFBP5 is concerned, Flu treatment had no effect on AI-induced IGFBP5 levels. These results suggest that T does not participate in the regulation of IGFBP5 expression in CL. However, additional studies are required to assess other effects of T on CL function.

The observations in the present study are one of the first reports on IGFBP5 and IGF action on ERα activity in CL that is mediated partly via PI3K/Akt pathway. In the CL, the prominent IGFBP5 mRNA expression has not been observed [6]. In the present study, a good association with changes in levels of E_2_ and expression of IGFBP5 in the CL was observed. Since changes in few of the molecules of PI3K/Akt signalling pathway were observed, it remains to be determined whether expression of IGFBP5 was regulated by the PI3K/Akt pathway. Earlier reports have suggested that E_2_ synthesized from placental androgens in the luteal cells cause hypertrophy of LLC population [12, 22]. Interestingly, mRNA expression for different members of IGF system show SLCs to be the major source of IGF1 and IGFBP3 and five, whereas, IGFBP2 and four are expressed approximately to same extent in both SLCs and LLCs [7]. The findings in the present study point to inhibition of hypertrophy of SLC, brought about by lack of E_2_ biosynthesis. This implies that E_2_ may affect IGF1 mRNA expression differently depending on whether the cells are proliferating. The interaction between E_2_ and growth factor signalling pathways has been well established [41, 44]. Additional studies are necessary to unravel whether regulation of CL function also involves interaction between E_2_ and IGF1 signalling. The additive or synergistic effects of IGF1 and E_2_ on cell proliferation, tumor development, anti-apoptosis and vascular protection have been well described [2, 37]. Based on the observations, it appears that the non-genomic signalling pathway activated by the phosphorylation of ERα induced by E_2_ gets inhibited in the presence of AI perhaps due to increased IGFBP5 expression [5]. PI3K/Akt pathway has been further documented in ERα stabilization and hormone dependent and independent ERα activation process [17, 27].

The findings of the present study together with the observations by others [26] suggest influence of IGF on ERα activity involving PI3K/Akt pathway. The findings from this study suggest inhibitory effect of IGFBP5 on E_2_ induced ERα function is perhaps by way of sequestration of IGF1. The increased expression of PI3K/Akt signal pathway genes is suggestive of increased anabolic metabolism, cell proliferation and survival [23, 38]. Finally, in the present study, the changes observed in the CL are associated with presence or absence of E_2_, but not by its substrate, androgen. Based on findings in this study and others, we propose a model for E_2_ actions and is represented in Fig. 8e.

## Conclusion

In conclusion, the role of E_2_ in the regulation of luteal steroidogenesis, hypertrophy and proliferation of cells in the luteal tissue was analysed, and IGFBP5 was identified as one of the E_2_ responsive genes that play an important role in the mediation of E_2_ action perhaps due to the E_2_-induced phosphorylation of PI3K/Akt pathway.

## Additional files

Additional file 1: Table S1. List of antibodies used for immunoblot analyses in rats. The list of antibodies employed in the immunoblot analyses including the target sequence, antigen sequence, catalog number, species raised in, dilution used, molecular weight and manufacturer’s name. (TIF 2107 kb) Additional file 2: Table S2. List of primers used for qPCR analyses. The list of genes and details of the primers employed along with the expected amplicon size and annealing temperature are provided. (PPTX 63 kb) Additional file 3: Figure S1. In vitro aromatisation of testosterone (T) and effects of AI on circulating E_2_ and P_4_ levels, weight of CL and *Cyp19a1* mRNA expression during rat pregnancy. (A) Sliced pieces of CL tissue collected on day 7, 11, 12 and 16 of rat pregnancy was incubated without or with 20 ng T for 4 h. E_2_ content in the medium was estimated and represented as pg/mg tissue/4 h. Each bar represents mean±SEM, *n* = 4 to 12/time point, ^***^ *P* <0.001, ^*^ *P* <0.05. (B-E) Pregnant rats received oral administration of AI (1 mg/kg BW) or VEH (2 % ethanol) for four days daily. Circulating mean±SEM serum E_2_ (B) and P_4_ (C) concentrations during different treatments (*n* = 5 to 10 animals/time point, B and *n* = 3 animals/time point, C, ^**^ *P* <0.01, ^*^ *P* <0.05). (E) Weight of CL during different treatments with a representative photo for each treatment is shown on each bar (mean±SEM, *n* = 8 to 13 CL/time point) (F) qPCR expression of *Cyp19a1* mRNA in CL post different treatments. The results are shown as fold changes of mRNA expression compared to day 7 CL. Individual bars represents mean±SEM fold change in mRNA expression value for qPCR analysis during different treatments (*n* = 4 animals/time point). (PPTX 338 kb) Additional file 4: Table S3. List of networks involved during E_2_inhibition using Ingenuity Pathway Analysis (IPA). IPA was used to classify the differentially expressed genes into different function and disease categories. IPA on the differentially expressed genes for each of the treatments examined and cross-talk and network overlapping were determined. Networks were identified with score and number of focus molecules ranging from as low as one to as high as 22 are represented. (PPTX 70 kb) Additional file 5: Table S4. List of networks involved during E_2_ replacement AI+E_2_ using Ingenuity Pathway Analysis (IPA). IPA on the differentially expressed genes for each of the treatments examined and cross-talk and network overlapping are studied during E_2_ replacement experiments are represented. (PPTX 72 kb) Additional file 6: Figure S2. Classification of differentially expressed genes associated with steroidogenesis and growth factors in response to AI treatment. IPA of the differentially regulated genes post AI treatment shows a network of 26 (steroidogenesis) and 16 (growth factors) focus molecules. The network is displayed graphically as nodes (gene/gene products) and edges (biological relationship between nodes). The node colour intensity indicates the fold change expression of genes with red representing up regulation and green, down regulation of genes. The shapes of nodes indicate the functional class of the gene product and the lines indicate the type of interaction. (PPTX 500 kb) Additional file 7: Table S5. List of top 15 up regulated E_2_ responsive genes post VEH or AI treatment. Potential E_2_ responsive genes in rat CL were identified based on the available list of classical E_2_ responsive genes, genes employed in PCR array human estrogen signalling and the data base, ERGDB. Microarray data analysis was carried out to obtain a set of differentially expressed genes based on statistics- Student’s t-test (two tail, unpaired) with *P* <0.05 and multiple hypothesis testing (Benjamini and Hochberg comparison test) to reduce the false positives. The identified differentially expressed E_2_ responsive genes were transcript consistent and did not hybridize to multiple transcripts, as suggested by the AffyProbeMiner analysis. A Bioconductor analysis was performed with ≤1.5 fold change as cut-off and statistical filters for identification of differentially expressed E_2_ responsive genes. Top 15 UP regulated E_2_ responsive genes post VEH or AI treatment are represented. Probe Set ID: The identifier that refers to a set of probe pairs selected to represent expressed sequences on an array; Gene symbol: Extracted from Entrez Gene or UniGene; Gene Title: Gene name extracted from Entrez Gene or UniGene. (PPTX 83 kb) Additional file 8: Table S6. List of top 15 up regulated E_2_ responsive genes post AI or AI+E_2_ treatment Top 15 UP regulated E_2_ responsive genes post AI or AI+E_2_ treatment are represented. (PPTX 85 kb)
