# Supplementary figures and images for: Analysis of 17β-estradiol (E2) role in the regulation of corpus luteum function in pregnant rats: Involvement of IGFBP5 in the E2-mediated actions
Source: Reprod Biol Endocrinol. 2016 Apr 12;14:19. doi: 10.1186/s12958-016-0153-1 (PMC4830059; doi:10.1186/s12958-016-0153-1)

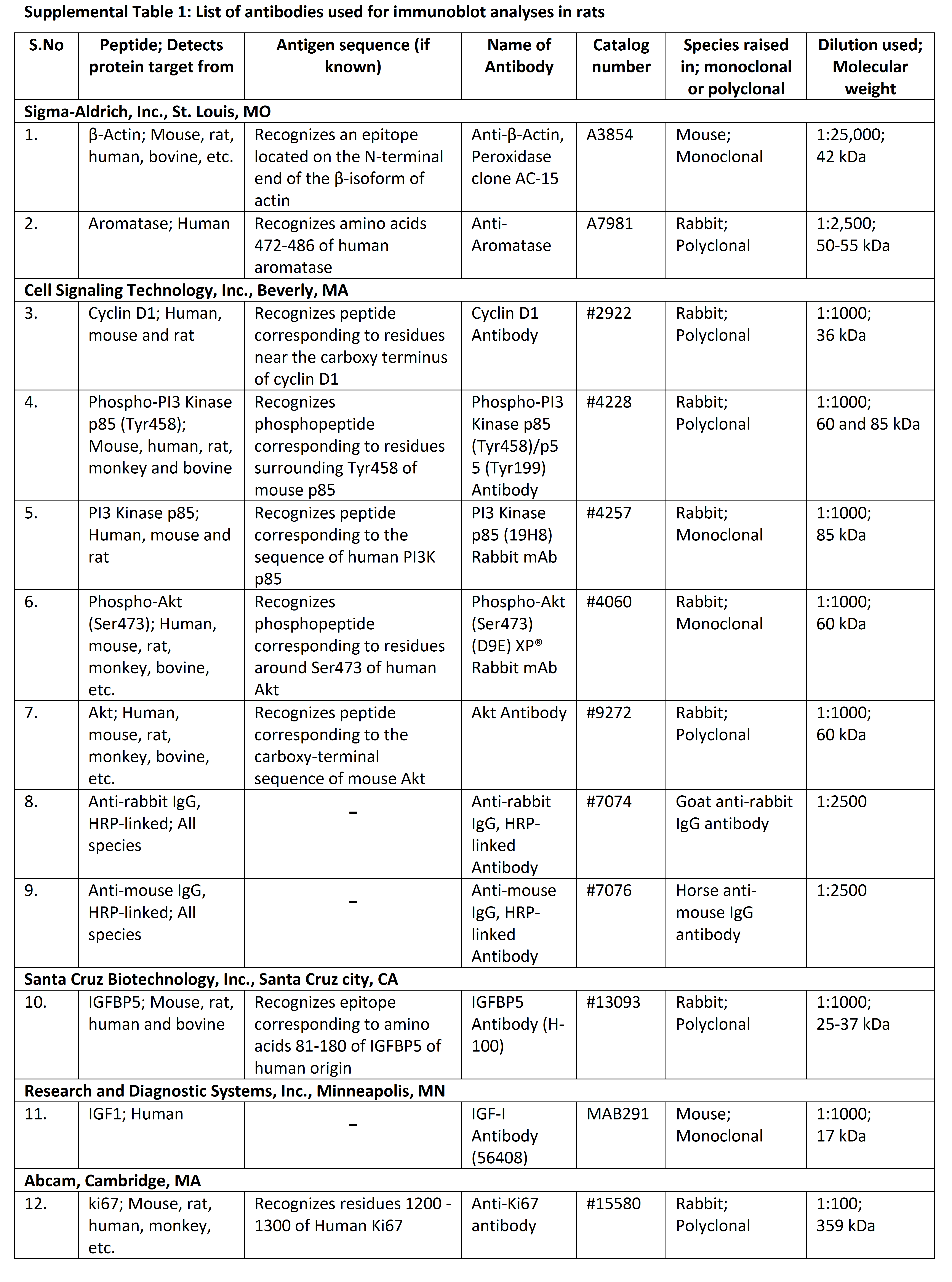

Supplement: Additional file 1: Table S1. — List of antibodies used for immunoblot analyses in rats. The list of antibodies employed in the immunoblot analyses including the target sequence, antigen sequence, catalog number, species raised in, dilution used, molecular weight and manufacturer’s name. (TIF 2107 kb) [file 12958_2016_153_MOESM1_ESM.tif]

## Slide 1
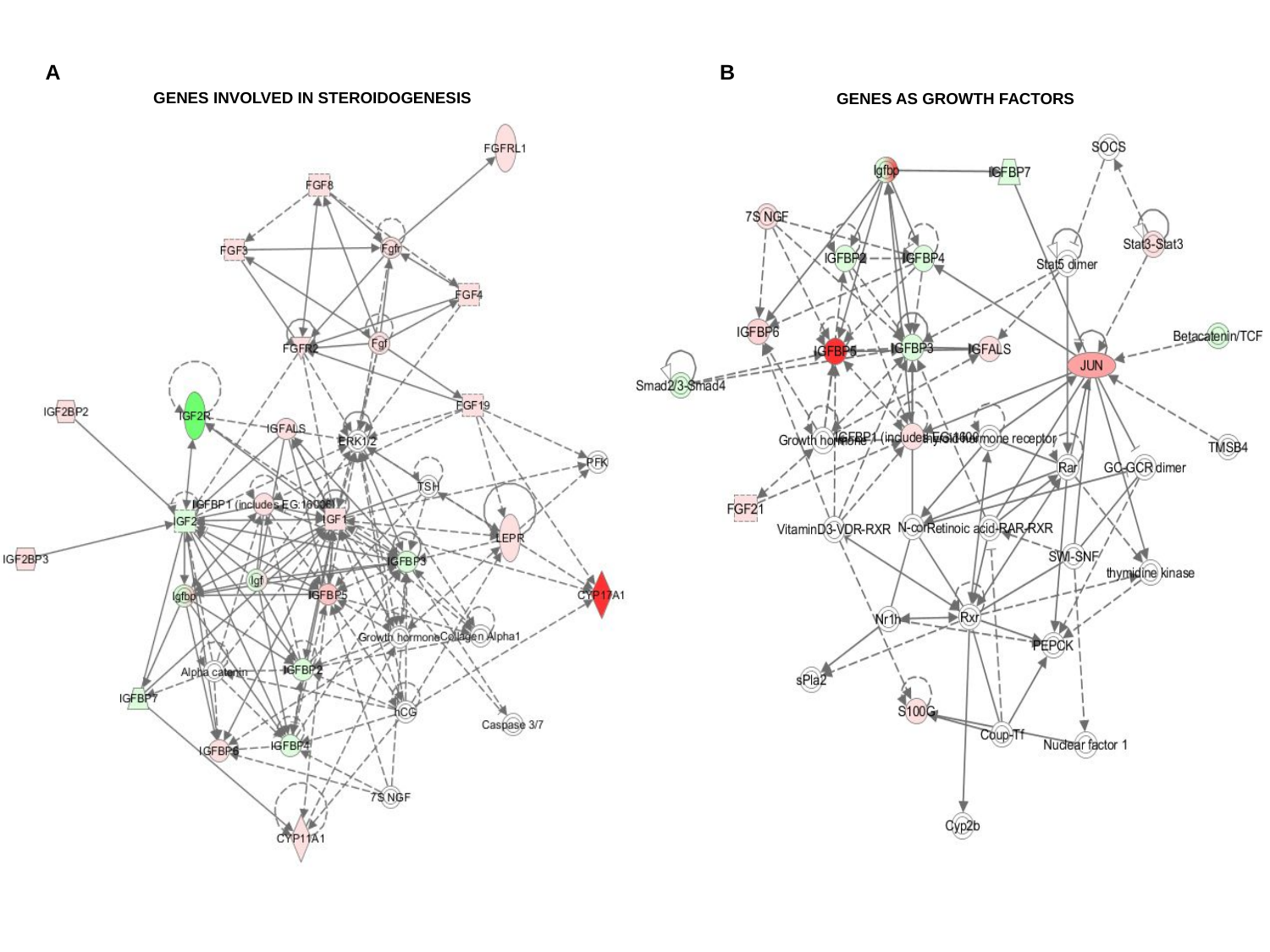

A
B
GENES INVOLVED IN STEROIDOGENESIS
GENES AS GROWTH FACTORS

Supplement: Additional file 6: Figure S2. — Classification of differentially expressed genes associated with steroidogenesis and growth factors in response to AI treatment. IPA of the differentially regulated genes post AI treatment shows a network of 26 (steroidogenesis) and 16 (growth factors) focus molecules. The network is displayed graphically as nodes (gene/gene products) and edges (biological relationship between nodes). The node colour intensity indicates the fold change expression of genes with red representing up regulation and green, down regulation of genes. The shapes of nodes indicate the functional class of the gene product and the lines indicate the type of interaction. (PPTX 500 kb) [file 12958_2016_153_MOESM6_ESM.pptx]
