# Supplementary material for: Analysis of 17β-estradiol (E2) role in the regulation of corpus luteum function in pregnant rats: Involvement of IGFBP5 in the E2-mediated actions
Source: Reprod Biol Endocrinol. 2016 Apr 12;14:19. doi: 10.1186/s12958-016-0153-1 (PMC4830059; doi:10.1186/s12958-016-0153-1)
Supplement: Additional file 2: Table S2. — List of primers used for qPCR analyses. The list of genes and details of the primers employed along with the expected amplicon size and annealing temperature are provided. (PPTX 63 kb) [file 12958_2016_153_MOESM2_ESM.pptx]

## Slide 1
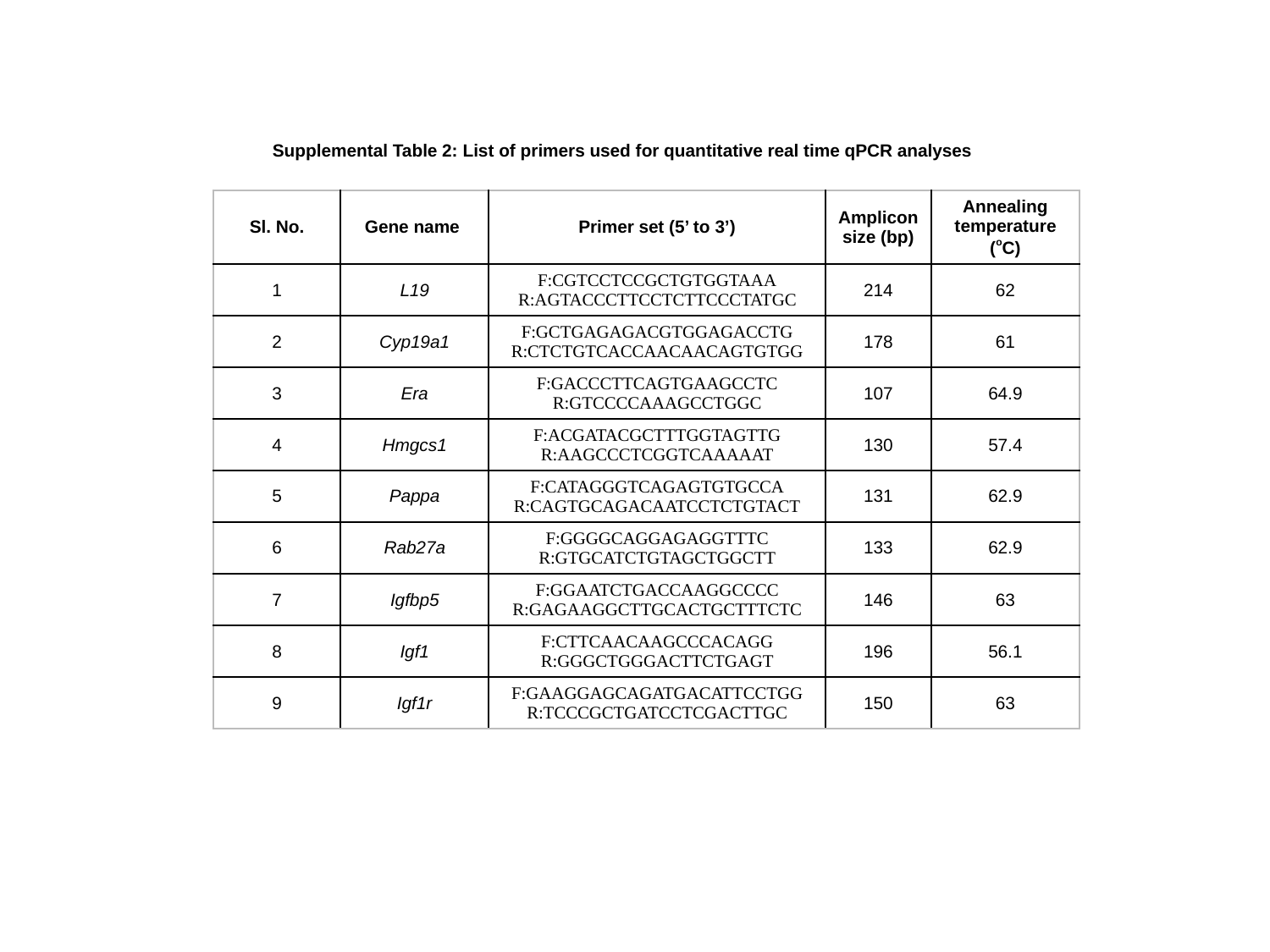

Supplemental Table 2: List of primers used for quantitative real time qPCR analyses
| Sl. No. | Gene name | Primer set (5’ to 3’) | Amplicon size (bp) | Annealing temperature (oC) |
| --- | --- | --- | --- | --- |
| 1 | L19 | F:CGTCCTCCGCTGTGGTAAA R:AGTACCCTTCCTCTTCCCTATGC | 214 | 62 |
| 2 | Cyp19a1 | F:GCTGAGAGACGTGGAGACCTG R:CTCTGTCACCAACAACAGTGTGG | 178 | 61 |
| 3 | Era | F:GACCCTTCAGTGAAGCCTC R:GTCCCCAAAGCCTGGC | 107 | 64.9 |
| 4 | Hmgcs1 | F:ACGATACGCTTTGGTAGTTG R:AAGCCCTCGGTCAAAAAT | 130 | 57.4 |
| 5 | Pappa | F:CATAGGGTCAGAGTGTGCCA R:CAGTGCAGACAATCCTCTGTACT | 131 | 62.9 |
| 6 | Rab27a | F:GGGGCAGGAGAGGTTTC R:GTGCATCTGTAGCTGGCTT | 133 | 62.9 |
| 7 | Igfbp5 | F:GGAATCTGACCAAGGCCCC R:GAGAAGGCTTGCACTGCTTTCTC | 146 | 63 |
| 8 | Igf1 | F:CTTCAACAAGCCCACAGG R:GGGCTGGGACTTCTGAGT | 196 | 56.1 |
| 9 | Igf1r | F:GAAGGAGCAGATGACATTCCTGG R:TCCCGCTGATCCTCGACTTGC | 150 | 63 |
