# Supplementary material for: Analysis of 17β-estradiol (E2) role in the regulation of corpus luteum function in pregnant rats: Involvement of IGFBP5 in the E2-mediated actions
Source: Reprod Biol Endocrinol. 2016 Apr 12;14:19. doi: 10.1186/s12958-016-0153-1 (PMC4830059; doi:10.1186/s12958-016-0153-1)
Supplement: Additional file 3: Figure S1. — In vitro aromatisation of testosterone (T) and effects of AI on circulating E2 and P4 levels, weight of CL and Cyp19a1 mRNA expression during rat pregnancy. (A) Sliced pieces of CL tissue collected on day 7, 11, 12 and 16 of rat pregnancy was incubated without or with 20 ng T for 4 h. E2 content in the medium was estimated and represented as pg/mg tissue/4 h. Each bar represents mean±SEM, n = 4 to 12/time point, *** P <0.001, * P <0.05. (B-E) Pregnant rats received oral administration of AI (1 mg/kg BW) or VEH (2 % ethanol) for four days daily. Circulating mean±SEM serum E2 (B) and P4 (C) concentrations during different treatments (n = 5 to 10 animals/time point, B and n = 3 animals/time point, C, ** P <0.01, * P <0.05). (E) Weight of CL during different treatments with a representative photo for each treatment is shown on each bar (mean±SEM, n = 8 to 13 CL/time point) (F) qPCR expression of Cyp19a1 mRNA in CL post different treatments. The results are shown as fold changes of mRNA expression compared to day 7 CL. Individual bars represents mean±SEM fold change in mRNA expression value for qPCR analysis during different treatments (n = 4 animals/time point). (PPTX 338 kb) [file 12958_2016_153_MOESM3_ESM.pptx]

## Slide 1
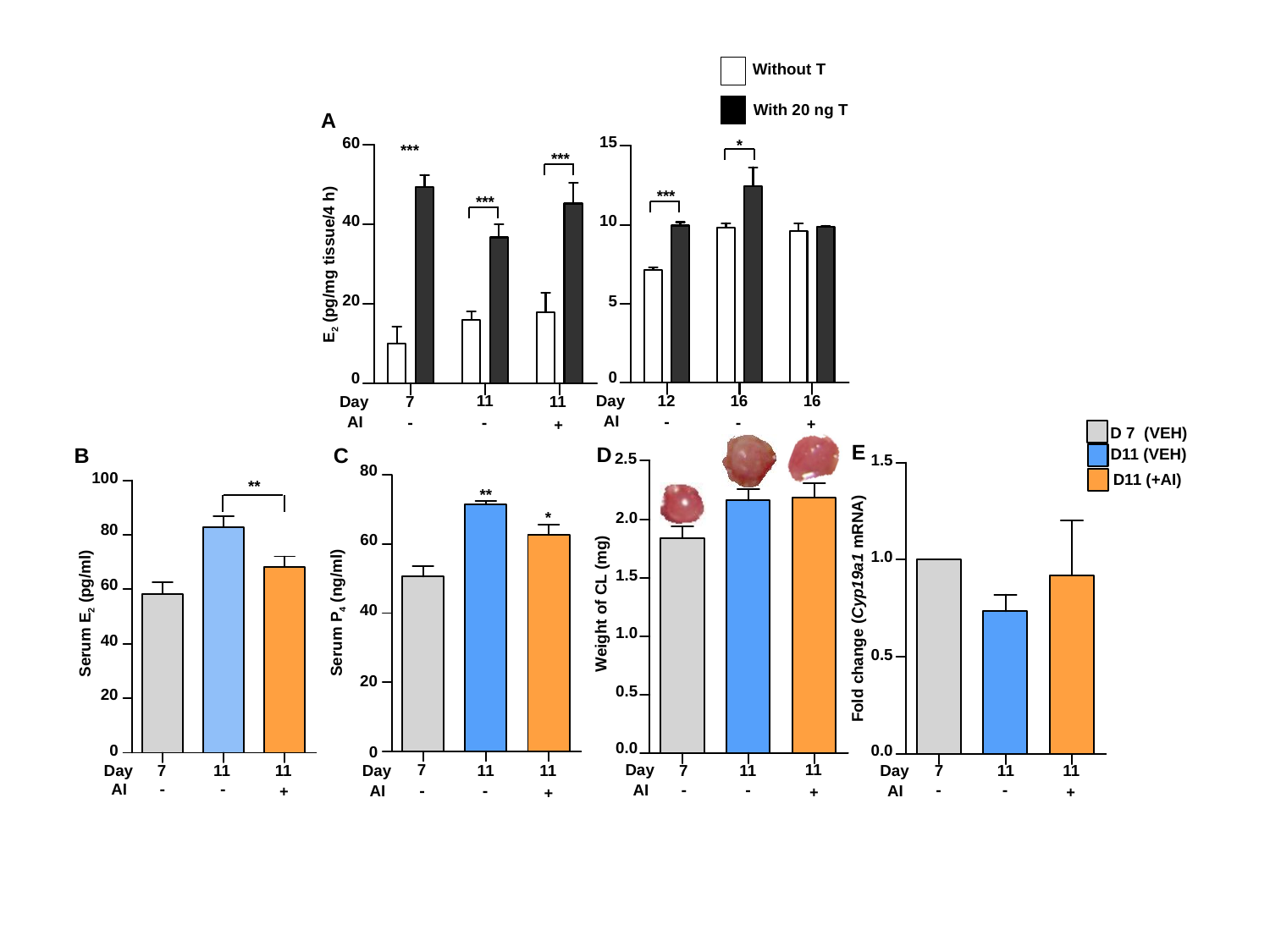

Without T
With 20 ng T
60
40
E2 (pg/mg tissue/4 h)
20
0
11
7
Day
11
AI
-
-
+
***
A
15
10
5
0
***
12
Day
16
16
AI
-
-
+
*
***
***
B
100
80
60
 Serum E2 (pg/ml)
40
20
0
11
Day
11
7
-
-
AI
+
**
E
1.5
1.0
Fold change (Cyp19a1 mRNA)
0.5
0.0
11
Day
11
7
-
-
AI
+
2.5
2.0
1.5
Weight of CL (mg)
1.0
0.5
0.0
D
Day
11
7
11
-
-
AI
+
80
60
40
Serum P4 (ng/ml)
20
0
C
7
11
Day
11
-
-
AI
+
**
*
D 7 (VEH)
D11 (VEH)
D11 (+AI)
