# Supplementary material for: Analysis of 17β-estradiol (E2) role in the regulation of corpus luteum function in pregnant rats: Involvement of IGFBP5 in the E2-mediated actions
Source: Reprod Biol Endocrinol. 2016 Apr 12;14:19. doi: 10.1186/s12958-016-0153-1 (PMC4830059; doi:10.1186/s12958-016-0153-1)
Supplement: Additional file 4: Table S3. — List of networks involved during E2inhibition using Ingenuity Pathway Analysis (IPA). IPA was used to classify the differentially expressed genes into different function and disease categories. IPA on the differentially expressed genes for each of the treatments examined and cross-talk and network overlapping were determined. Networks were identified with score and number of focus molecules ranging from as low as one to as high as 22 are represented. (PPTX 70 kb) [file 12958_2016_153_MOESM4_ESM.pptx]

## Slide 1
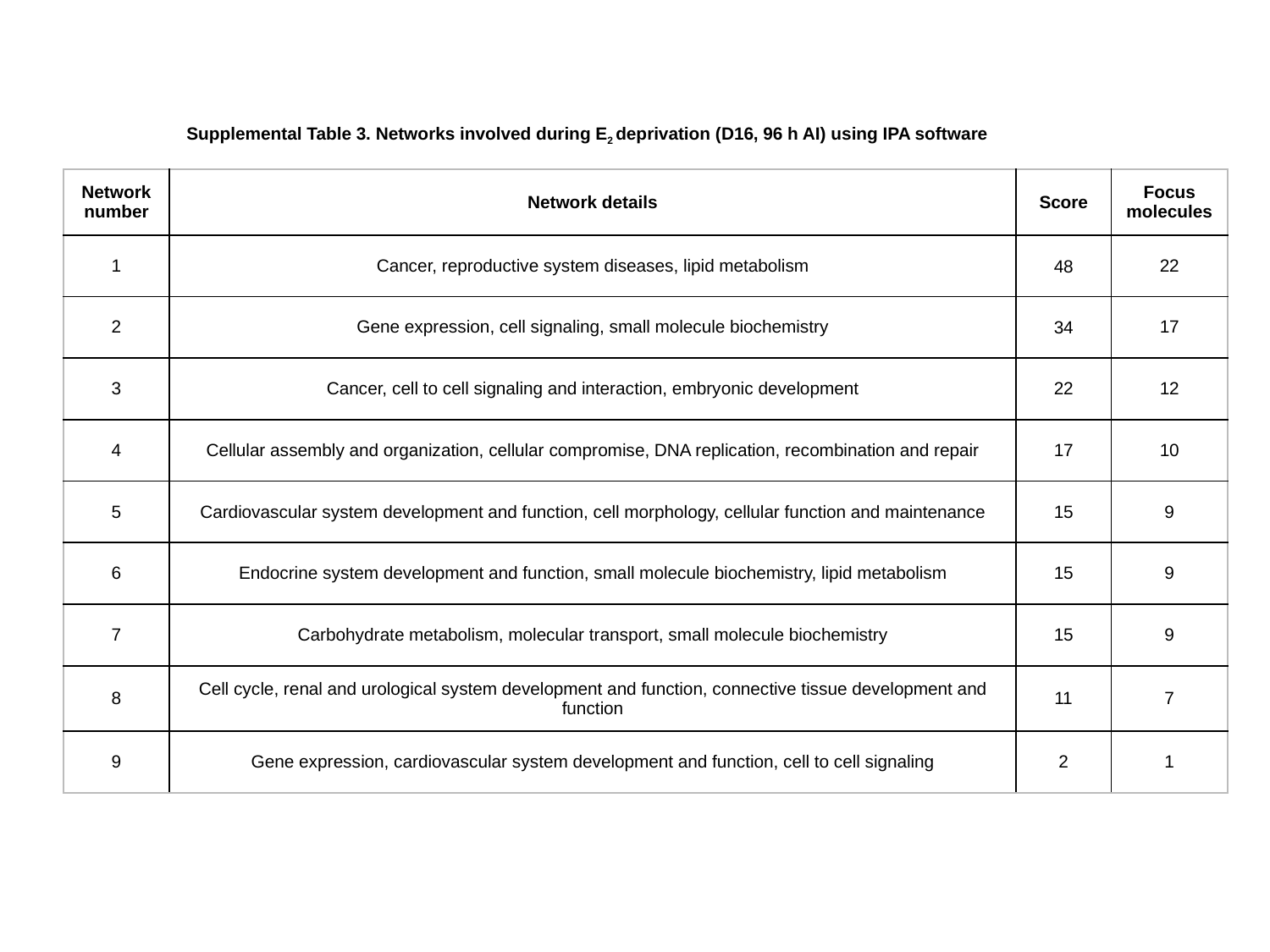

Supplemental Table 3. Networks involved during E2 deprivation (D16, 96 h AI) using IPA software
| Network number | Network details | Score | Focus molecules |
| --- | --- | --- | --- |
| 1 | Cancer, reproductive system diseases, lipid metabolism | 48 | 22 |
| 2 | Gene expression, cell signaling, small molecule biochemistry | 34 | 17 |
| 3 | Cancer, cell to cell signaling and interaction, embryonic development | 22 | 12 |
| 4 | Cellular assembly and organization, cellular compromise, DNA replication, recombination and repair | 17 | 10 |
| 5 | Cardiovascular system development and function, cell morphology, cellular function and maintenance | 15 | 9 |
| 6 | Endocrine system development and function, small molecule biochemistry, lipid metabolism | 15 | 9 |
| 7 | Carbohydrate metabolism, molecular transport, small molecule biochemistry | 15 | 9 |
| 8 | Cell cycle, renal and urological system development and function, connective tissue development and function | 11 | 7 |
| 9 | Gene expression, cardiovascular system development and function, cell to cell signaling | 2 | 1 |
