# Supplementary material for: Analysis of 17β-estradiol (E2) role in the regulation of corpus luteum function in pregnant rats: Involvement of IGFBP5 in the E2-mediated actions
Source: Reprod Biol Endocrinol. 2016 Apr 12;14:19. doi: 10.1186/s12958-016-0153-1 (PMC4830059; doi:10.1186/s12958-016-0153-1)
Supplement: Additional file 5: Table S4. — List of networks involved during E2 replacement AI+E2 using Ingenuity Pathway Analysis (IPA). IPA on the differentially expressed genes for each of the treatments examined and cross-talk and network overlapping are studied during E2 replacement experiments are represented. (PPTX 72 kb) [file 12958_2016_153_MOESM5_ESM.pptx]

## Slide 1
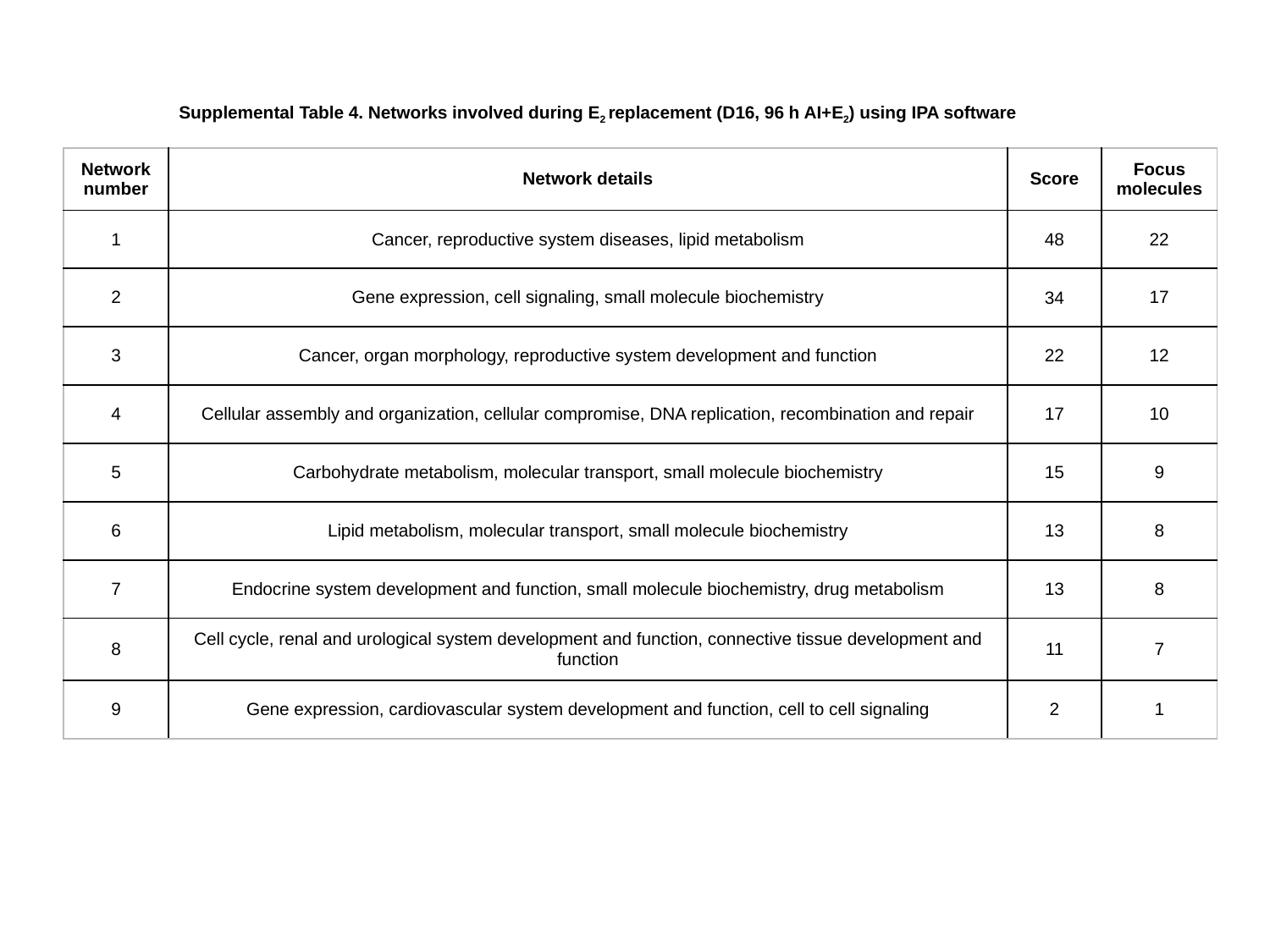

Supplemental Table 4. Networks involved during E2 replacement (D16, 96 h AI+E2) using IPA software
| Network number | Network details | Score | Focus molecules |
| --- | --- | --- | --- |
| 1 | Cancer, reproductive system diseases, lipid metabolism | 48 | 22 |
| 2 | Gene expression, cell signaling, small molecule biochemistry | 34 | 17 |
| 3 | Cancer, organ morphology, reproductive system development and function | 22 | 12 |
| 4 | Cellular assembly and organization, cellular compromise, DNA replication, recombination and repair | 17 | 10 |
| 5 | Carbohydrate metabolism, molecular transport, small molecule biochemistry | 15 | 9 |
| 6 | Lipid metabolism, molecular transport, small molecule biochemistry | 13 | 8 |
| 7 | Endocrine system development and function, small molecule biochemistry, drug metabolism | 13 | 8 |
| 8 | Cell cycle, renal and urological system development and function, connective tissue development and function | 11 | 7 |
| 9 | Gene expression, cardiovascular system development and function, cell to cell signaling | 2 | 1 |
