# Supplementary material for: Analysis of 17β-estradiol (E2) role in the regulation of corpus luteum function in pregnant rats: Involvement of IGFBP5 in the E2-mediated actions
Source: Reprod Biol Endocrinol. 2016 Apr 12;14:19. doi: 10.1186/s12958-016-0153-1 (PMC4830059; doi:10.1186/s12958-016-0153-1)
Supplement: Additional file 7: Table S5. — List of top 15 up regulated E2 responsive genes post VEH or AI treatment. Potential E2 responsive genes in rat CL were identified based on the available list of classical E2 responsive genes, genes employed in PCR array human estrogen signalling and the data base, ERGDB. Microarray data analysis was carried out to obtain a set of differentially expressed genes based on statistics- Student’s t-test (two tail, unpaired) with P <0.05 and multiple hypothesis testing (Benjamini and Hochberg comparison test) to reduce the false positives. The identified differentially expressed E2 responsive genes were transcript consistent and did not hybridize to multiple transcripts, as suggested by the AffyProbeMiner analysis. A Bioconductor analysis was performed with ≤1.5 fold change as cut-off and statistical filters for identification of differentially expressed E2 responsive genes. Top 15 UP regulated E2 responsive genes post VEH or AI treatment are represented. Probe Set ID: The identifier that refers to a set of probe pairs selected to represent expressed sequences on an array; Gene symbol: Extracted from Entrez Gene or UniGene; Gene Title: Gene name extracted from Entrez Gene or UniGene. (PPTX 83 kb) [file 12958_2016_153_MOESM7_ESM.pptx]

## Slide 1
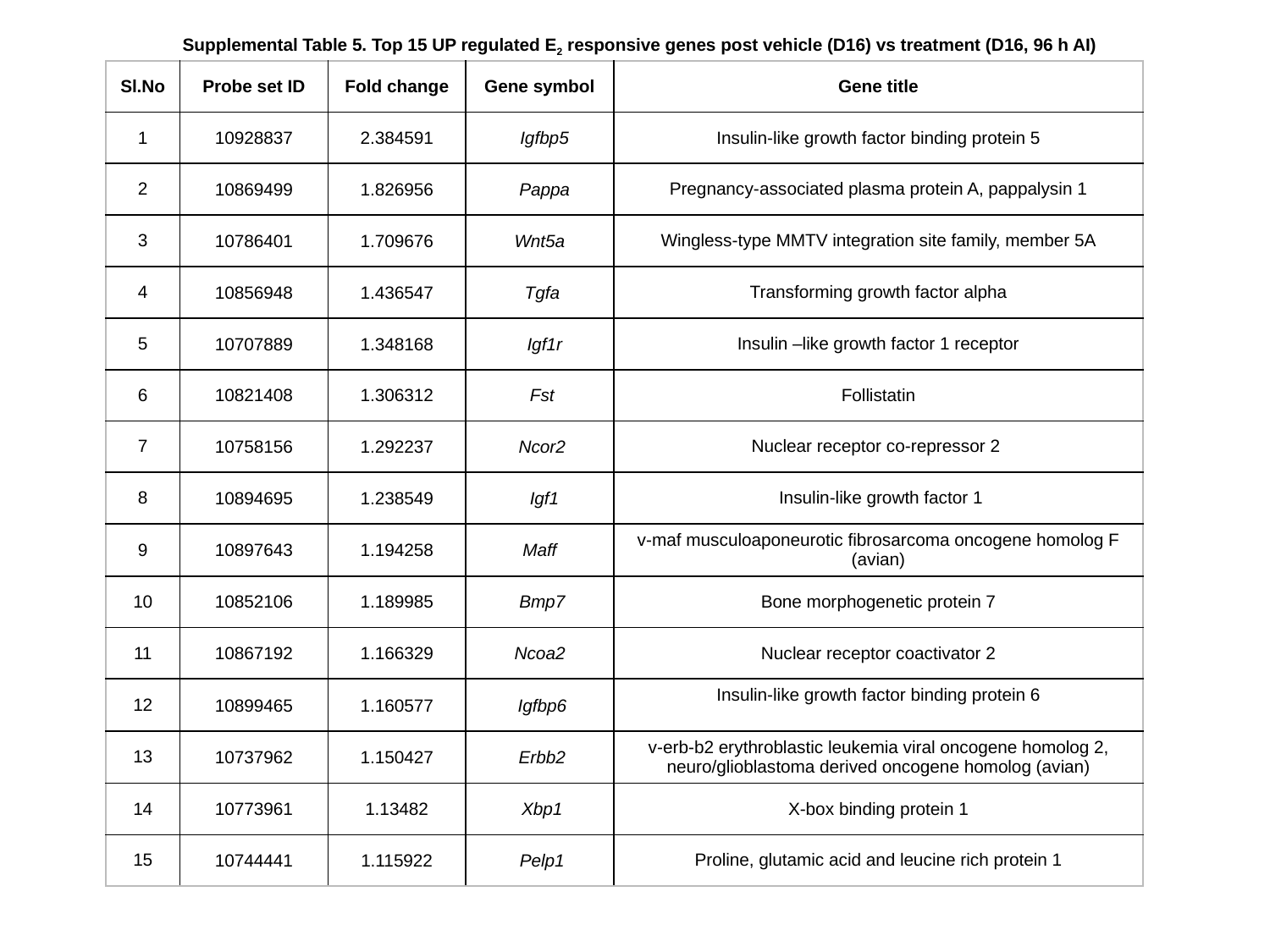

Supplemental Table 5. Top 15 UP regulated E2 responsive genes post vehicle (D16) vs treatment (D16, 96 h AI)
| Sl.No | Probe set ID | Fold change | Gene symbol | Gene title |
| --- | --- | --- | --- | --- |
| 1 | 10928837 | 2.384591 | Igfbp5 | Insulin-like growth factor binding protein 5 |
| 2 | 10869499 | 1.826956 | Pappa | Pregnancy-associated plasma protein A, pappalysin 1 |
| 3 | 10786401 | 1.709676 | Wnt5a | Wingless-type MMTV integration site family, member 5A |
| 4 | 10856948 | 1.436547 | Tgfa | Transforming growth factor alpha |
| 5 | 10707889 | 1.348168 | Igf1r | Insulin –like growth factor 1 receptor |
| 6 | 10821408 | 1.306312 | Fst | Follistatin |
| 7 | 10758156 | 1.292237 | Ncor2 | Nuclear receptor co-repressor 2 |
| 8 | 10894695 | 1.238549 | Igf1 | Insulin-like growth factor 1 |
| 9 | 10897643 | 1.194258 | Maff | v-maf musculoaponeurotic fibrosarcoma oncogene homolog F (avian) |
| 10 | 10852106 | 1.189985 | Bmp7 | Bone morphogenetic protein 7 |
| 11 | 10867192 | 1.166329 | Ncoa2 | Nuclear receptor coactivator 2 |
| 12 | 10899465 | 1.160577 | Igfbp6 | Insulin-like growth factor binding protein 6 |
| 13 | 10737962 | 1.150427 | Erbb2 | v-erb-b2 erythroblastic leukemia viral oncogene homolog 2, neuro/glioblastoma derived oncogene homolog (avian) |
| 14 | 10773961 | 1.13482 | Xbp1 | X-box binding protein 1 |
| 15 | 10744441 | 1.115922 | Pelp1 | Proline, glutamic acid and leucine rich protein 1 |
