# Supplementary material for: Analysis of 17β-estradiol (E2) role in the regulation of corpus luteum function in pregnant rats: Involvement of IGFBP5 in the E2-mediated actions
Source: Reprod Biol Endocrinol. 2016 Apr 12;14:19. doi: 10.1186/s12958-016-0153-1 (PMC4830059; doi:10.1186/s12958-016-0153-1)
Supplement: Additional file 8: Table S6. — List of top 15 up regulated E2 responsive genes post AI or AI+E2 treatment Top 15 UP regulated E2 responsive genes post AI or AI+E2 treatment are represented. (PPTX 85 kb) [file 12958_2016_153_MOESM8_ESM.pptx]

## Slide 1
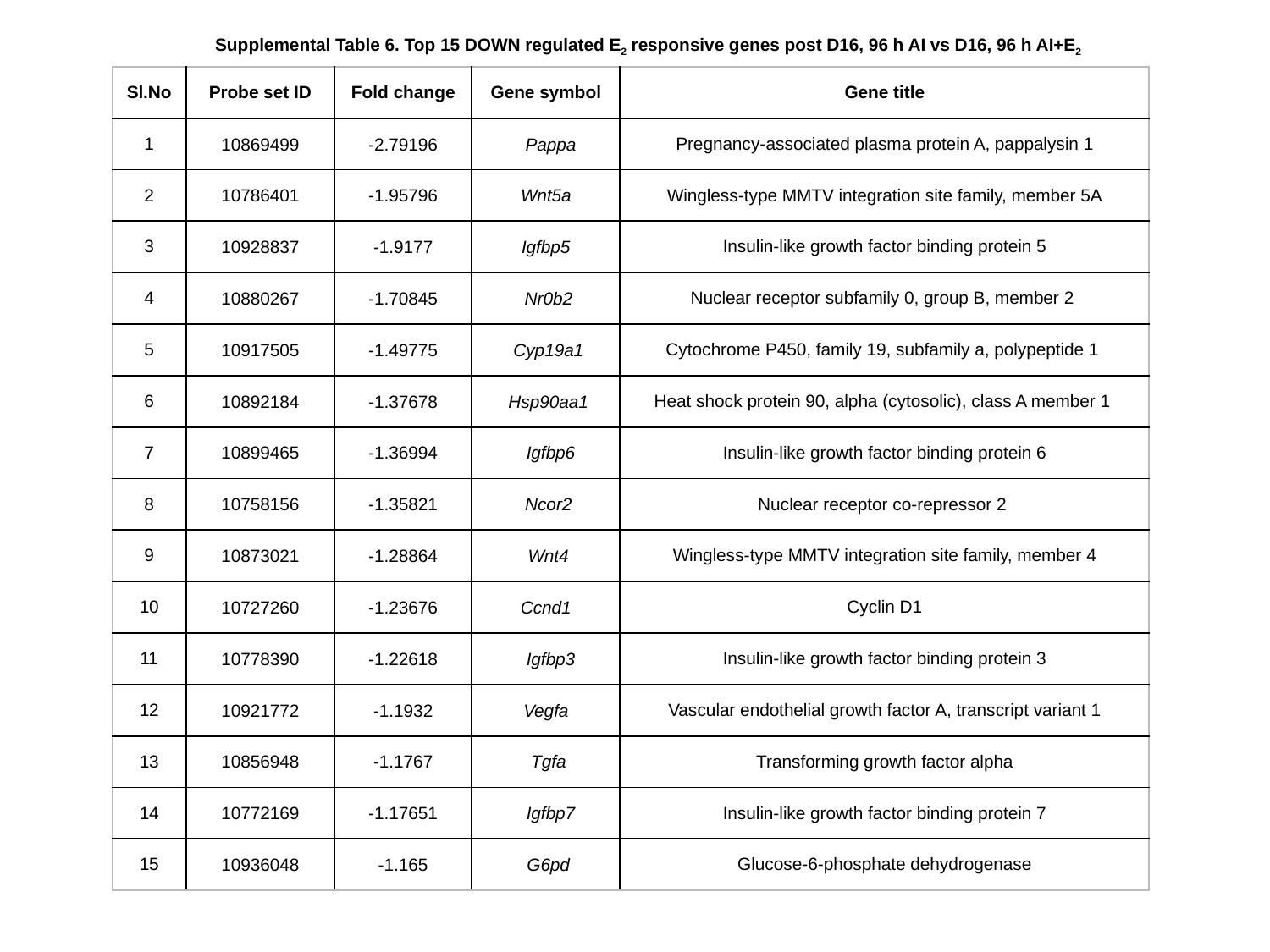

Supplemental Table 6. Top 15 DOWN regulated E2 responsive genes post D16, 96 h AI vs D16, 96 h AI+E2
| Sl.No | Probe set ID | Fold change | Gene symbol | Gene title |
| --- | --- | --- | --- | --- |
| 1 | 10869499 | -2.79196 | Pappa | Pregnancy-associated plasma protein A, pappalysin 1 |
| 2 | 10786401 | -1.95796 | Wnt5a | Wingless-type MMTV integration site family, member 5A |
| 3 | 10928837 | -1.9177 | Igfbp5 | Insulin-like growth factor binding protein 5 |
| 4 | 10880267 | -1.70845 | Nr0b2 | Nuclear receptor subfamily 0, group B, member 2 |
| 5 | 10917505 | -1.49775 | Cyp19a1 | Cytochrome P450, family 19, subfamily a, polypeptide 1 |
| 6 | 10892184 | -1.37678 | Hsp90aa1 | Heat shock protein 90, alpha (cytosolic), class A member 1 |
| 7 | 10899465 | -1.36994 | Igfbp6 | Insulin-like growth factor binding protein 6 |
| 8 | 10758156 | -1.35821 | Ncor2 | Nuclear receptor co-repressor 2 |
| 9 | 10873021 | -1.28864 | Wnt4 | Wingless-type MMTV integration site family, member 4 |
| 10 | 10727260 | -1.23676 | Ccnd1 | Cyclin D1 |
| 11 | 10778390 | -1.22618 | Igfbp3 | Insulin-like growth factor binding protein 3 |
| 12 | 10921772 | -1.1932 | Vegfa | Vascular endothelial growth factor A, transcript variant 1 |
| 13 | 10856948 | -1.1767 | Tgfa | Transforming growth factor alpha |
| 14 | 10772169 | -1.17651 | Igfbp7 | Insulin-like growth factor binding protein 7 |
| 15 | 10936048 | -1.165 | G6pd | Glucose-6-phosphate dehydrogenase |
